# Synergistic curative effects of *Trichoderma hamatum* and *Rumex dentatus* against *Alternaria alternata*, the causal agent of tomato leaf spot disease

**DOI:** 10.3389/fpls.2025.1700051

**Published:** 2025-12-09

**Authors:** Bassant Philip, Asia R. Eid, Shokry R. Bayoumi, Ahmed Heflish, Osama O. Atallah, Eman A. Abdelwahab, Monika Michalecka, Said I. Behiry, Ahmed Abdelkhalek, Abdulaziz A. Al-Askar

**Affiliations:** 1 Agricultural Botany Department, Faculty of Agriculture (Saba Basha), Alexandria University, Alexandria, Egypt; 2 Plant Pathology Department, Faculty of Agriculture, Damanhour University, El-Beheira, Egypt; 3 Division of Genetics, Faculty of Agriculture, Damanhour University, El-Beheira, Egypt; 4 Plant Pathology Department, Faculty of Agriculture, Zagazig University, Zagazig, Egypt; 5 College of Physical Education and Sport Sciences, Al-Mustaqbal University, Babylon, Iraq; 6 Plant Protection Department, The National Institute of Horticultural Research, Skierniewice, Poland; 7 Plant Protection and Biomolecular Diagnosis Department, Arid Lands Cultivation Research Institute, City of Scientific Research and Technological Applications, Alexandria, Egypt; 8 Department of Botany and Microbiology, College of Science, King Saud University, Riyadh, Saudi Arabia

**Keywords:** *Alternaria alternata*, tomato, antioxidant enzymes, disease severity, GC-MS, HPLC

## Abstract

The current investigation identified and characterized *Alternaria alternata* as the causal agent of tomato leaf spot through morphological traits and multilocus phylogenetic analysis [internal transcribed spacer (ITS), translation elongation factor 1-alpha (*tef1*-α), and the RNA polymerase II second-largest subunit (*RPB2*)], thereby confirming its classification within the *A. alternata* complex. Four *Trichoderma* isolates (Ham34, Ham35, Ham36, and Ham37) were recovered from loamy rhizosphere soil associated with healthy tomato plants. Among them, Ham34 (*Trichoderma hamatum*) showed the most significant antagonistic activity in dual-culture assays. Ethyl acetate extracts of all isolates exhibited concentration-dependent antifungal effects, with Ham34 showing the most potent inhibition (57.8% at 2000 µg/mL). Gas chromatography-mass spectrometry (GC-MS) profiling of the Ham34 extract identified bioactive metabolites, including (-)-spathulenol (28.2%) and glycerol 1,2-diacetate (21.4%). Among the four tested plant extracts, *Rumex dentatus* (RD) showed superior activity, achieving 68.5% inhibition at 2000 µg/mL, followed by moderate inhibition from *Cichorium intybus*, *Conium maculatum*, and *Capsicum annuum*. High-performance liquid chromatography (HPLC) profiling of *Rumex dentatus* extract revealed high levels of phenolic and flavonoid compounds, with a total concentration of 108.63 µg/mL. The primary identified constituents were rutin (33.1 µg/mL), gallic acid (22.7 µg/mL), and chlorogenic acid (17.9 µg/mL). Under greenhouse conditions, tomato plants (*Solanum lycopersicum* cv. Super Strain B) were treated with Ham34 ethyl acetate extract, RD extract, and their combination (Ham34 + RD). The combined treatment significantly reduced disease incidence (11.1%) and severity (16.7%), outperforming Ridomil Gold^®^ in disease suppression. Growth parameters and chlorophyll content (SPAD) were significantly enhanced in the combined treatment, with increased shoot and root biomass and length. Biochemical analyses revealed that the combined Ham34 + RD treatment enhanced antioxidant enzyme activities, including catalase (CAT) and peroxidase (POD), and increased total soluble protein content to 342.8 µg/mL compared to 331.1 µg/mL in the untreated control. Moreover, the treatment elevated total phenolic content to 256 mg GAE/g, indicating improved stress mitigation and enhanced plant vigor. This eco-friendly approach provides a low-cost, chemically stable, and safe alternative to synthetic fungicides, highlighting the synergistic potential of integrating *R. dentatus* and *T. hamatum* for sustainable management of *A. alternata*-induced tomato leaf spot.

## 1 Introduction

Tomato (*Solanum lycopersicum*) is a globally significant agricultural crop, widely cultivated for its nutritional value and economic importance (Echeverría-Pérez et al., 2025). It serves as a staple in fresh consumption and various food processing industries worldwide. One of the most prevalent and destructive fungal diseases affecting tomato plants is leaf spot, primarily caused by the necrotrophic pathogen *Alternaria alternata* (Bhushan et al., 2025). This pathogen leads to significant economic losses by compromising plant health, reducing photosynthetic efficiency, and ultimately diminishing fruit quality and quantity (Yang et al., 2018; Gupta and Saxena, 2023; Wei et al., 2025). Traditional disease management strategies heavily rely on synthetic chemical fungicides (Ul Haq et al., 2020; Akhtar et al., 2024). While effective in controlling the disease, these chemical interventions raise considerable environmental and health concerns, including soil and water contamination, pesticide residues in food products, and the development of fungicide resistance in pathogen populations (Beckerman et al., 2023; Cech et al., 2023; Ahmad et al., 2024). Consequently, there is an urgent need to develop and implement ecologically friendly and sustainable disease management strategies for tomato cultivation.

In response to this critical need, research has increasingly focused on exploring natural plant-derived compounds and beneficial microorganisms as viable biocontrol agents (El-Saadony et al., 2022; Ayaz et al., 2023; Tyagi et al., 2024). *Rumex dentatus* (Polygonaceae), a plant traditionally recognized for its medicinal properties, has shown promising antifungal activity in preliminary studies (Zhu et al., 2006; Humeera et al., 2013; Khaliq et al., 2023). Its efficacy is attributed to a rich profile of polyphenolic compounds, such as rutin, gallic acid, and chlorogenic acid, which possess well-documented antimicrobial properties (Shahab Khan and Ahmad, 2022). *Cichorium intybus* (Asteraceae) has demonstrated antifungal activity, particularly root extracts containing sesquiterpene lactones (e.g., 8-deoxylactucin and 11β,13-dihydrolactucin), which have shown growth inhibition and morphological alterations in dermatophytic fungi such as *Trichophyton tonsurans* (Mares et al., 2005). Additional investigations reveal that the water and ethyl acetate fractions of *C. intybus* are active against *Fusarium solani* and *Aspergillus niger* (Rehman et al., 2014). *Conium maculatum* (*Apiaceae*), though toxic, produces inducible furanocoumarins (e.g., isopimpinellin, umbelliferone, xanthotoxin) that significantly increase in response to stress (e.g., CuCl_2_ elicitation) and exhibit potent antifungal activity, particularly in *Conium* leaves (Al-Barwani and Eltayeb, 2004). *Capsicum annuum* (*Solanaceae*) offers multiple antifungal compounds. The thionin-like peptide CaThi, isolated from chili peppers, exhibits potent candidacidal activity through membrane permeabilization, induction of oxidative stress, and synergism with fluconazole against various *Candida* species (Taveira et al., 2016). Additionally, capsaicinoids (e.g., capsaicin) exhibit antifungal effects against pathogens such as *Botrytis cinerea*, *Aspergillus flavus*, and *Fusarium oxysporum*, primarily by disrupting fungal membranes and inducing osmotic stress (Costa et al., 2022). At the same time, rhizospheric *Trichoderma* species are becoming increasingly recognized for their effectiveness as biological control agents and promoters of plant growth (Philip et al., 2024). The antagonistic effects of these fungi on plant pathogens are achieved through several mechanisms, such as mycoparasitism, the production of antifungal metabolites, and the induction of systemic resistance in host plants (Awad-Allah et al., 2022; Zehra et al., 2023). Furthermore, *Trichoderma* isolates are known to enhance nutrient uptake and promote overall plant growth, thereby improving plant vigor and resilience (Rodríguez-Martínez et al., 2025).

Recent studies have highlighted the synergistic potential of *Trichoderma* when combined with other biological or chemical agents, leading to enhanced disease suppression and plant defense activation (Li et al., 2020; Abd-Elsalam et al., 2025; Kumari et al., 2025). For instance, the co-application of *Trichoderma harzianum* with paclobutrazol significantly improved tomato seedling performance and reduced pathogen impact under greenhouse conditions (Shalaby et al., 2022). Similarly, co-cultures of *Trichoderma* and *Burkholderia vietnamiensis* bacteria have been reported to increase secondary metabolite production and antifungal efficacy (Li et al., 2024). *Trichoderma* and *Azotobacter* co-cultivation has demonstrated mutual compatibility, resulting in enhanced biomass, biofilm formation, and the production of antifungal metabolites (Velmourougane et al., 2017). In agricultural systems, synergistic microbial consortia comprising *Trichoderma*, *Bacillus*, *Burkholderia*, and *Pseudomonas* have demonstrated remarkable potential as biocontrol agents, as they can utilize their complementary traits to enhance plant growth and disease suppression (Poveda and Eugui, 2022). These synergistic strategies not only amplify biocontrol effectiveness but also reduce the required dosages of individual agents, offering a cost-efficient and environmentally sustainable approach (Hafiz et al., 2022; Wang et al., 2024).

Molecular-based approaches, especially multilocus sequence analysis (MLSA), have become essential tools for accurate fungal species identification, as morphological traits often lack sufficient specificity (O’Donnell et al., 2008; Cai et al., 2009; Stielow et al., 2015). The internal transcribed spacer (ITS) region is considered the universal fungal barcode and serves as the primary marker in most identification workflows (Schocha et al., 2012). However, ITS alone may not provide the resolution needed in closely related taxa, such as *Alternaria* and *Trichoderma*. To enhance discriminatory power, protein-coding genes such as translation elongation factor 1-alpha (*tef1*-α) and the RNA polymerase II second-largest subunit (*RPB2*) are commonly used as complementary markers. These loci offer superior single-copy variability and phylogenetic depth across fungal genera (Větrovský et al., 2016; Lücking et al., 2020). For *Trichoderma* taxonomy, ITS, *tef1*, and *RPB2* are standard markers utilized in multilocus databases and identification systems such as MIST (Dou et al., 2020; Jambhulkar et al., 2024). Similarly, combined ITS–*tef1*–*RPB2* sequence datasets have been widely applied to resolve species boundaries within both *Trichoderma* and *Alternaria* through robust phylogenetic reconstruction (Raja et al., 2017; Nuangmek et al., 2021). This multilocus strategy, integrating both ribosomal (ITS) and protein-coding (*tef1*, *RPB2*) genes, corresponds with current best practices in fungal systematics (MLSA), ensuring precise and reproducible species identification.

Plants, in turn, possess intricate defense mechanisms to counteract biological stresses, such as fungal infections. A key component of this defense system involves the activation of antioxidant enzymes, such as catalase (CAT), peroxidase (POD), superoxide dismutase (SOD), and polyphenol oxidase (PPO) (Haghpanah et al., 2025; Sood, 2025). These enzymes play a crucial role in scavenging reactive oxygen species (ROS) that accumulate during stress, thereby mitigating oxidative damage (Patanè et al., 2022). The levels of malondialdehyde (MDA), a marker of lipid peroxidation, and hydrogen peroxide (H_2_O_2_), a signaling molecule and precursor to ROS, are often indicative of the extent of oxidative stress (Abdelkhalek et al., 2025a). Additionally, total protein (TP) and total phenolic compounds (TPC) contribute significantly to the plant’s defense response and overall immunity (Chowdhary et al., 2021). The present study evaluates the efficacy of *R. dentatus* extracts and *Trichoderma* isolates, particularly *T. hamatum*, against *A. alternata*-induced leaf spot in tomato plants under greenhouse conditions. Unlike previous studies focusing on single control agents, this work integrates plant extracts with fungal biocontrol to reveal their synergistic effects and the associated antioxidant and biochemical defense responses in tomato leaves. The study is driven by the need for safer, low-cost alternatives to chemical fungicides, as both *R. dentatus* and *Trichoderma* can be produced from readily available natural resources, offering an economical and environmentally sustainable disease management strategy.

## 2 Materials and methods

### 2.1 Isolation and morphological characterization of the leaf spot pathogen

Tomato leaves showing characteristic symptoms of leaf spot were collected from *S. lycopersicum* L. cv. ‘Super Strain B’, one of the commonly cultivated commercial varieties in Egypt, is grown under open-field conditions in El-Beheira Governorate, Egypt (30°53’02.2”N, 30°41’04.6”E). Sampling was performed during the 2023 growing season, at the mid-fruiting stage (approximately 75 days after transplanting) when disease symptoms were clearly visible. A total of 20 symptomatic plants were randomly selected. Of these, 30 leaf samples were processed for pathogen isolation and morphological characterization. Fully expanded secondary and tertiary leaves from the middle portion of symptomatic plants were selected to ensure active infection and minimize senescence-related artifacts. Leaves were collected in sterile polyethylene bags and transported to the laboratory under chilled conditions for further analysis. Infected leaf segments (approximately 5 mm²) were excised from the advancing margins of lesions, avoiding severely necrotic tissue. Samples were surface-sterilized by immersion in 70% ethanol for 30 seconds, followed by immersion in a 1% sodium hypochlorite solution for 1 minute, and then rinsed three times with sterile distilled water. The sterilized segments were then blot-dried using sterile filter paper and aseptically plated onto Potato Dextrose Agar (PDA) medium supplemented with 100 µg/mL streptomycin to suppress bacterial growth (Agrios, 2005; Behiry et al., 2022b). Plates were incubated at 25 ± 2°C for 7 days in the dark using an incubator (Model: TITANOX S.r.l., Cremona, Italy). Emerging fungal colonies were sub-cultured onto fresh PDA plates using hyphal tip transfer to obtain pure cultures. Morphological characteristics of colonies exhibiting typical *Alternaria* features, including colony color, growth rate, margin shape, and spore morphology, were recorded (Barnett and Hunter, 1998). Microscopic characteristics were analyzed using lactophenol-cotton blue-stained specimens under a compound light microscope at 400× magnification.

### 2.2 Isolation and morphological characterization of *Trichoderma*

Rhizospheric soil samples were collected from the same tomato cultivar ‘Super Strain B’ grown in healthy, disease-free fields in El-Beheira Governorate, Egypt (30°51’46.4”N, 30°41’58.4”E). The plants were approximately 60 days old, corresponding to the flowering-to-early fruit-setting stage, when rhizosphere microbial activity is at its peak. Soil adhering closely to the roots (0–3 mm) was considered rhizospheric soil and carefully collected at a depth of 10–15 cm using a sterile trowel. Approximately 200 g of soil was transferred into sterile polyethylene bags, labeled, and transported to the laboratory for immediate processing (Aseel et al., 2023). Ten grams of each soil sample was suspended in 90 mL of sterile distilled water and shaken vigorously for 30 min. Serial dilutions were prepared up to 10^-5^, and 0.1 mL aliquots from 10^-^³ to 10^-5^ dilutions were plated onto Rose Bengal Agar (RBA) and PDA media supplemented with streptomycin sulfate (100 µg/mL) to inhibit bacterial contamination (Castle et al., 1998; Gil et al., 2009). Plates were incubated at 25 ± 2°C for 7 days. Morphologically distinct colonies exhibiting typical *Trichoderma* features were purified and characterized. Colony morphology, including color, texture, margin, and growth rate, was documented. Microscopic features were examined using lactophenol cotton blue-stained preparations under a compound light microscope at 400× magnification.

### 2.3 PCR amplification and molecular characterization of *Alternaria* sp.

Fungal genomic DNA was extracted from 7-day-old mycelia grown in Potato Dextrose Broth (PDB) using a modified cetyltrimethylammonium bromide (CTAB) method (Soliman et al., 2023). For *Alternaria* sp. identification, three DNA regions were amplified: the ITS region of ribosomal DNA (rDNA) using primers ITS1 (5′-TCCGTAGGTGAACCTGCGG-3′) and ITS4 (5′-TCCTCCGCTTATTGATATGC-3′) (White et al., 1990); the *tef1* gene using primers *EF1*-728F (5′-CATCGAGAAGTTCGAGAAGG-3′) and *EF1*-986R (5′-TACTTGAAGGAACCCTTACC-3′) (Carbone and Kohn, 1999); and the *RPB2* gene using primers f*RPB2*-5F (5′-GAYGAYMGWGATCAYTTYGG-3′) and f*RPB2*-7cR (5′-CCCATRGCTTGYTTRCCCAT-3′) (Liu et al., 1999). PCR amplification was performed in 25 µL reaction mixtures that included 1× PCR buffer, 2.0 mM MgCl_2_, 0.2 mM of each dNTP, 0.4 µM of each primer, 1 unit of Taq DNA polymerase, and approximately 50 ng of template DNA. The thermocycling parameters for ITS included an initial denaturation at 94°C for 5 minutes, followed by 35 cycles comprising denaturation at 94°C for 30 seconds, annealing at 55°C for 30 seconds, and extension at 72°C for 1 minute, concluding with a final extension at 72°C for 10 minutes. The annealing temperatures for *tef1* amplification and *RPB2* were set at 58°C and 54°C, respectively. PCR products were resolved on 1.2% agarose gels, stained with ethidium bromide, and visualized using UV light. The resultant sequences were compared with those in the NCBI GenBank database via BLASTn, and the annotated sequences were submitted to the GenBank database.

### 2.4 Multilocus sequence analysis and phylogenetic tree construction of *Alternaria* sp.

Given the limitations of one-gene sequence analysis in reliably determining species affiliation (Behiry et al., 2023), a multilocus sequence analysis (MLSA) was performed. The evolutionary analyses were performed using MEGA12 (Kumar et al., 2024), which employs up to four parallel computational threads. MEGA12 employs the Bayesian Information Criterion (BIC) and the corrected Akaike Information Criterion (AICc) to assess model fit, with these criteria computed from the log-likelihood fit of each model to the specified multiple sequence alignment and its corresponding parameters (Tamura et al., 2011). Based on the lowest BIC (36804.870) and AICc (36438.629) thresholds, the evolutionary rate variance among sites was modeled using a Kimura 2-parameter model with a discrete Gamma distribution (+*G*) comprising five rate categories (Yang, 1994), while permitting a percentage of sites to be evolutionarily invariant (+*I*). The maximum-likelihood (ML) phylogenetic tree was derived from a concatenation of the three genes [ITS (516 bp), RPB2 (1074 bp), and *tef1* (904 bp)] of *Alternaria* sp. strain Alt2, along with 19 type strains within the genus *Alternaria*. The *Fusarium oxysporum* strain Fo47^T^, a member of the genus *Fusarium*, was used as an outgroup taxon for phylogenetic inference. The ML analyses were performed utilizing an automatically generated tree topology (Tamura et al., 2011). The partial deletion option was used to remove all positions with less than 95% site coverage, resulting in a final dataset of 2,239 positions. The bootstrap analysis employed the standard option in MEGA12 with 500 replicates, while the adaptive option utilized a default standard error level of 5%.

### 2.5 Effect of *Trichoderma* isolates against the growth of the *Alternaria* pathogen *in vitro*

The antagonistic potential of the isolated *Trichoderma* sp. isolates against the *Alternaria* sp. leaf spot pathogen was evaluated *in vitro* using two approaches: the dual-culture technique and the application of ethyl acetate extracts. For the dual-culture assay, 5-mm mycelial discs of each *Trichoderma* isolate were placed on one side (approximately 1 cm from the edge) of a PDA plate. In contrast, similar discs of the *Alternaria* pathogen were placed on the opposite side. Control plates were inoculated with *Alternaria* sp. alone. The plates were incubated at 25 ± 2 °C for 7 days, and the radial growth of the pathogen was measured. Percent inhibition of radial growth (PIRG) was calculated using the formula: PIRG (%) = [(R1 − R2)/R1] × 100, where R1 is the radial growth of *Alternaria* sp. in the control plates, and R2 is the growth in the presence of *Trichoderma* (Dennis and Webster, 1971). The preparation of the ethyl acetate extract involved extracting 500 mL of each *Trichoderma* sp. culture broth three times with equal volumes of ethyl acetate, using a separating funnel. The organic layers were combined and evaporated to dryness using a rotary evaporator set at 40°C. The residues obtained were dissolved in 10% dimethyl sulfoxide (DMSO) and subsequently incorporated into molten PDA to reach final concentrations of 0, 100, 500, 1000, and 2000 µg/mL. PDA plates were inoculated with discs of *Alternaria* sp. and incubated as described above. Growth inhibition was calculated similarly. Control plates were prepared with PDA containing 10% DMSO without any extract.

### 2.6 Molecular identification and phylogenetic construction of the most potent *Trichoderma* isolate

The selected Trichoderma isolate was subjected to genomic DNA extraction, PCR amplification, and sequencing for ITS, *tef1*, and *RPB2* genes, as previously described in sections 2.3 and 2.4. The annotated sequences were submitted to GenBank for accession number approval, and a phylogenetic tree was constructed using MEGA12 with 500 replicates (Kumar et al., 2024). Based on the lowest BIC (63592.733) and AICc (63130.120) thresholds, the evolutionary rate variance among sites was modeled using the Kimura 2-parameter model with +G+*I*. The maximum-likelihood (ML) phylogenetic tree was derived from a concatenation of the three genes [ITS (586 bp), RPB2 (1082 bp), and tef1 (1296 bp)] of *Trichoderma* sp. isolate Ham34, along with 24 type strains within the genus *Trichoderma*. The *Aspergillus flavus* strain NRRL 3357^T^, a member of the genus *Aspergillus*, was used as an outgroup taxon for phylogenetic inference. The partial deletion option was used to remove all positions with less than 95% site coverage, resulting in a final dataset of 2,521 positions.

### 2.7 GC-MS analysis of ethyl acetate extract of *Trichoderma* culture broth

An analysis using gas chromatography-mass spectrometry (GC-MS) was conducted to identify the bioactive constituents present in the ethyl acetate extract of the most effective antimicrobial *Trichoderma* isolate. The concentrated extract was filtered through a 0.22 µm syringe filter before analysis. The analysis was performed using an Agilent 7890A gas chromatograph (Agilent Technologies, Santa Clara, CA, USA) in conjunction with an Agilent 5975C mass selective detector. The system used an HP-5MS capillary column (30 m × 0.25 mm i.d., 0.25 µm film thickness), with helium as the carrier gas at a constant flow rate of 1.0 mL/min. The injection volume was set at 1 µL using splitless mode. The oven temperature was initially set to 50°C for 2 minutes, then increased to 150°C at a rate of 10°C per minute, and finally to 280°C at a rate of 5 °C per minute, where it was maintained for 10 minutes. The temperatures of the injector and detector were set to 250°C and 280°C, respectively. The mass spectrometer functioned in electron ionization (EI) mode at 70 eV, utilizing a scan range of 50–600 m/z. Compounds were identified by comparing their mass spectra with those in Wiley Registry 8e and the mainlib mass spectral libraries. Only peaks exhibiting a similarity index of 90% or greater were taken into account. The calculation of the relative abundance for each compound was performed using peak area normalization, with results expressed as a percentage of the total ion chromatogram (Abdelkhalek et al., 2022b).

### 2.8 Evaluation of plant extracts against the leaf spot pathogen

Four plant species, *R. dentatus* (RD), *C. intybus* (CI), *C. maculatum* (CM), and *C. annuum* (CA), were selected for their potential antifungal activity. Fresh plant materials (leaves and aerial parts) were collected, washed thoroughly with tap water, and surface-sterilized using 70% ethanol for 30 seconds. After drying at room temperature in the shade for 7–10 days, depending on the species, until a constant weight was achieved. The variation in drying duration reflects differences in leaf thickness and moisture content among plants, ensuring complete drying without degrading heat-sensitive bioactive compounds. The dried samples were then ground into a fine powder using a sterile electric grinder. Ethanolic extracts were prepared by soaking 50 g of powdered plant material in 500 mL of 95% ethanol for 72 hours with intermittent shaking. The extracts were filtered through Whatman No. 1 filter paper and subsequently concentrated using a rotary evaporator under reduced pressure at 40 °C to yield crude extracts (Sobhy et al., 2023). Using the poisoned food technique, the antifungal activity of the plant extracts was assessed *in vitro* against the isolated leaf spot pathogen. PDA medium was amended with each plant extract at final concentrations of 0, 100, 500, 1000, and 2000 µg/mL. The chemical fungicide Ridomil Gold^®^ (containing Metalaxyl-M (4%) and Mancozeb (64%)), manufactured by Syngenta, Germany, was used as a positive control at the same concentrations. A 5 mm mycelial disc of the actively growing margin of the pathogen culture was placed at the center of each plate. Plates were incubated at 25 ± 2°C for 7 days. Fungal growth inhibition was assessed by measuring the radial growth (colony diameter) and calculating the percentage inhibition using the formula: Inhibition (%) = [(C – T)/C] × 100, where C is the colony diameter in the control plate, and T is the diameter in the treatment plate (Grover and Moore, 1962). All treatments were conducted in triplicate. Control treatments included PDA plates with 0 µg/mL of plant extract or fungicide. The assay results were performed using a completely randomized design (CRD) and statistically analyzed using one-way ANOVA followed by the LSD test at a significance level of *p* ≤ 0.05 using Costat software version 6.303.

### 2.9 HPLC analysis of phenolic and flavonoid compounds of the plant extract

High-performance liquid chromatography (HPLC) was used to identify and quantify the phenolic and flavonoid compounds present in the most effective ethanolic plant extract, which demonstrated antifungal activity. The initial step involved filtering the crude extract using 0.45 µm syringe filters before injection. The analysis was performed using a Waters 2695 HPLC system equipped with a photodiode array detector and a reverse-phase C18 column (250 mm × 4.6 mm, 5 µm particle size). The mobile phase consisted of two solvents: solvent A (0.1% formic acid in water) and solvent B (acetonitrile). The gradient elution profile was as follows: from 0 to 5 minutes, 5% B; from 5 to 25 minutes, 5 to 25% B; from 25 to 40 minutes, 25 to 50% B; from 40 to 50 minutes, 50 to 100% B; and finally, a 10-minute re-equilibration period. The flow rate was set at 1.0 mL/min, with an injection volume of 20 µL. Detection occurred at 280 nm for phenolic acids and at 330 nm for flavonoids (Abdelkhalek et al., 2023). The quantification process involved a comparative analysis of retention times and UV spectra of sample peaks against a set of 19 authentic standards. These compounds included gallic acid, chlorogenic acid, catechin, methyl gallate, caffeic acid, syringic acid, pyrocatechol, rutin, ellagic acid, coumaric acid, vanillin, ferulic acid, naringenin, daidzein, quercetin, cinnamic acid, apigenin, kaempferol, and hesperidin. Standard curves were constructed for each compound over a concentration range of 10 to 200 µg/mL. The concentration of each compound in the plant extracts was then calculated based on the area under the curve (AUC) using external standard calibration. Results were expressed as µg/mL (Rabie et al., 2024).

### 2.10 Effect of the combination of plant extract and ethyl acetate extract of *Trichoderma* on the mycelial growth of *Alternaria* isolate

To assess potential synergistic effects, the most effective plant extract and the most active ethyl acetate extract of *Trichoderma*, previously identified *in vitro*, were combined and evaluated against *A. alternata* using the poisoned food technique. Stock solutions of each extract were prepared in sterile distilled water containing 10% DMSO. Equal volumes of both extracts were mixed at a 1:1 (v/v) ratio to obtain the combination treatment. The mixture was incorporated into molten PDA to achieve final extract concentrations of 100, 500, 1000, and 2000 µg/mL. PDA plates containing 10% DMSO without any extract served as the negative control, while plates amended with Ridomil Gold^®^ fungicide (2000 µg/mL) served as the positive control. Each treatment was replicated three times. A 5-mm diameter mycelial disc of *Alternaria* (7-day-old culture) was placed at the center of each plate, and plates were incubated at 25 ± 2°C for 7 days. The radial growth of the pathogen was measured, and the percentage of mycelial growth inhibition was calculated as previously described in 2.4 (Grover and Moore, 1962). An analysis of variance (ANOVA) was conducted on the obtained data, and the LSD test was used to compare means, with a significance threshold of *p* < 0.05.

### 2.11 Greenhouse evaluation of *Trichoderma* and plant extract against *Alternaria* leaf spot on tomato

A greenhouse experiment was conducted during 2024 at the Plant Pathology Research Greenhouse, Faculty of Agriculture Saba Basha, Alexandria University, Alexandria, Egypt, to evaluate the efficacy of assess the effectiveness of a selected *Trichoderma* isolate (Ham34) bio-product (ethyl acetate extract), an ethanolic extract of RD, and the fungicide Ridomil Gold^®^ (Metalaxyl-M + Mancozeb) against *Alternaria* leaf spot disease in tomato. Healthy tomato seedlings (4–5 weeks old, cv. ‘Super Strain B’) were transplanted into plastic pots (25 cm diameter) filled with sterilized loam soil composed of sand, silt, and clay in a 2:2:1 ratio (v/v/v). The soil mixture provided balanced aeration, drainage, and nutrient availability under greenhouse conditions maintained at 25 ± 2°C, 60–70% relative humidity, and a 12 h photoperiod. A preliminary experiment was conducted to optimize pathogen inoculum, treatment concentrations, and timing. After optimization, the whole greenhouse experiment was performed using a completely randomized design (CRD) with six treatments and three independent biological replicates per treatment. The treatments were established to evaluate the efficacy of different biological and chemical agents against *Alternaria* leaf spot in tomato plants. The treatments were as follows: healthy untreated control plants (B1); plants inoculated with *Alternaria* pathogen only (B2); plants sprayed with plant extract (RD) 48 hours after *Alternaria* inoculation (B3); plants treated with ethyl acetate extract of *Trichoderma* isolate Ham34–48 hours after pathogen inoculation (B4); plants treated with a mixture of RD extract and Ham34 ethyl acetate extract 48 hours after inoculation (B5) and plants treated with Ridomil Gold^®^ fungicide 48 hours after *Alternaria* inoculation (B6). All treatments were monitored for disease progression, symptom severity, and physiological responses. For pathogen inoculation, a fresh conidial suspension of *Alternaria* (1 × 10^6^ spores/mL) was prepared from a 10-day-old culture grown on PDA. The surface of the culture was gently flooded with 10 mL of sterile distilled water containing 0.01% Tween-20, and the conidia were dislodged by lightly scraping the colony surface with a sterile glass rod. The resulting suspension was filtered through two layers of sterile muslin cloth to remove mycelial fragments. The spore concentration was determined using a Neubauer hemocytometer and adjusted to 1 × 10^6^ spores/mL with sterile distilled water before inoculation. Plants were sprayed with 20 mL of conidial suspension (1 × 10^6^ spores/mL) per plant using a hand-held atomizer until runoff, ensuring uniform coverage of the leaf surfaces. After inoculation, plants were covered with transparent polyethylene bags for 48 hours to maintain high humidity and promote infection (Agrios, 2005). The ethyl acetate extract of Ham34 and the RD extract were applied by foliar spraying at a concentration of 2000 µg/mL, using 10 mL of extract solution per plant. Ridomil Gold^®^ was used at the recommended field rate (2 g/L). Disease severity was assessed 15 days post-inoculation using a 0–5 disease rating scale, where 0 indicated no symptoms and 5 indicated more than 75% of the leaf area infected or defoliated. The disease index (%) was calculated using the following formula: Disease Index (%) = [Σ (disease scale × number of infected leaves)]/([total leaves × maximum disease scale]) × 100 (Gomez and Gomez, 1984). Plant growth parameters (plant height, fresh and dry weight) were also recorded.

### 2.12 Biochemical analysis of tomato leaves

To evaluate the biochemical response of tomato plants to various treatments under *Alternaria* stress, several antioxidant enzyme activities, oxidative stress markers, and total biochemical content were measured from leaf samples collected 10 days post-inoculation. Fresh leaf tissues (0.5 g) were homogenized in 5 mL of 50 mM phosphate buffer (pH 7.0) with 1% (w/v) polyvinylpyrrolidone (PVP) at 4°C. The homogenate underwent centrifugation at 12,000 × g for 20 minutes at 4°C, after which the supernatant was used for enzymatic assays. The activity of POD was assessed using the method established by Hammerschmidt et al (Hammerschmidt et al., 1982), which involved monitoring the oxidation of guaiacol at 470 nm in the presence of H_2_O_2_. The activity of SOD was assessed by its capacity to inhibit the photochemical reduction of nitroblue tetrazolium (NBT), following the methodology outlined by Giannopolitis and Ries (Giannopolitis and Ries, 1977). The activity of CAT was evaluated by measuring the breakdown of H_2_O_2_, as indicated by a decrease in absorbance at 240 nm, using the method described by Aebi (Aebi, 1984). The activity of polyphenol oxidase (PPO) was evaluated using the method established by Mayer et al (Mayer et al., 1966), by monitoring the increase in absorbance at 495 nm due to the oxidation of catechol. Lipid peroxidation was assessed by measuring malondialdehyde (MDA) levels using the thiobarbituric acid reactive substances (TBARS) method, as described by Heath and Packer (Heath and Packer, 1968). The titanium chloride method, as defined by Velikova et al (Velikova et al., 2000), was employed to determine the amount of hydrogen peroxide (H_2_O_2_). The quantification of total phenolic content was conducted utilizing the Folin–Ciocalteu reagent method (Singleton and Rossi, 1965), with absorbance readings taken at 765 nm. The total soluble protein content was determined using the Bradford method, with bovine serum albumin (BSA) as the standard. Fresh leaf tissue (0.5 g) from each treatment was homogenized in 5 mL of 50 mM phosphate buffer (pH 7.0) and centrifuged at 10,000 rpm for 10 min at 4°C. The supernatant was collected, and 100 µL was mixed with 5 mL of Bradford reagent. After 10 min, absorbance was recorded at 595 nm using a spectrophotometer. Protein concentration was determined from a BSA standard curve and expressed as µg/mL of extract (Bradford, 1976). All spectrophotometric measurements were conducted using a UV–VIS spectrophotometer (Thermo Scientific Genesys 10S UV-Vis, USA), and enzyme activities were expressed as μM/g FW. Each assay was performed in triplicate.

### 2.13 Statistical analysis

All experiments were conducted using three independent biological replicates, each measured with an appropriate number of technical replicates. Before statistical analysis, data were tested for normality using the Shapiro–Wilk test and for homogeneity of variances using Levene’s test. Since all datasets satisfied the assumptions of ANOVA, raw (non-transformed) data were used. Statistical analyses were performed using CoStat software version 6.303 (CoHort Software, Monterey, CA, USA). One-way ANOVA was used to evaluate treatment effects, and mean separations were determined using the least significant difference (LSD) test at a significance level of *p* ≤ 0.05. Results are expressed as the mean ± standard deviation (SD), and the coefficient of variation (CV%) was calculated for each treatment.

## 3 Results

### 3.1 Isolation and characterization of the leaf spot pathogen

**Figure 1:**
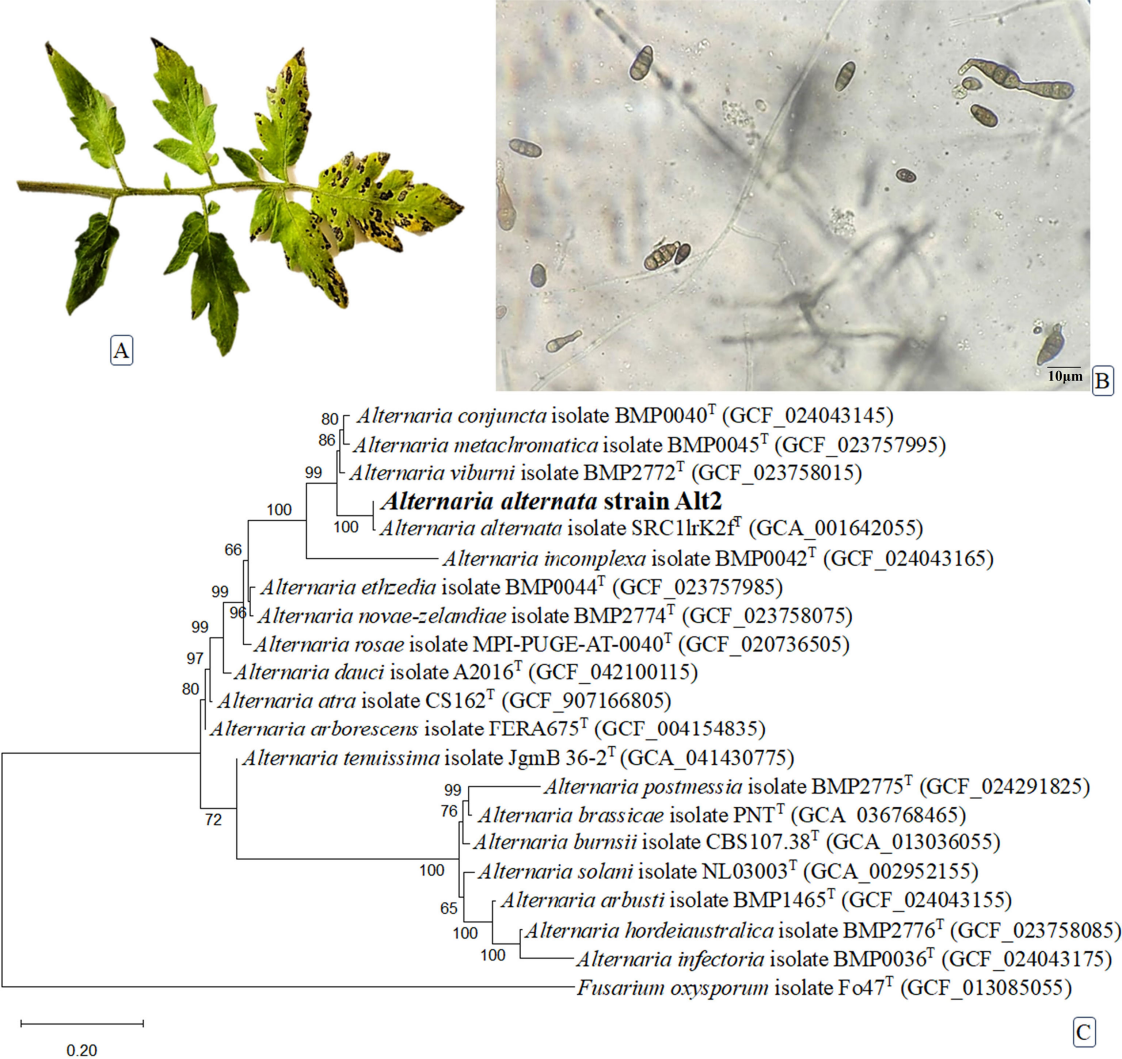
Leaf spot symptoms on infected tomato plants **(A)***Alternaria* microscopic conidia with both transverse and longitudinal septa. Scale bar = 10µm **(B)** the maximum-likelihood phylogenetic tree illustrates the relationship of the *Alternaria alternata* strain Alt2 (in bold) with other type strains within the genus *Alternaria*, derived from a concatenation of three genes: ITS, *RPB2*, and *tef1***(C)**. *Fusarium oxysporum*, a member of the genus *Fusarium*, was used as an outgroup taxon for phylogenetic inference. The partial deletion method was used to remove all positions with less than 95% site coverage, resulting in a final dataset of 2,239 positions. Bootstrap values were derived from 500 replicates using the optimal model (Kimura 2-parameter +*G* +*I*) in MEGA12 software, which utilized up to four parallel computing threads. The tree was rooted and scaled based on branch lengths, with the scale bar representing 0.2 substitutions per site. Type strains (T) are indicated in superscript.

Leaf spot symptoms were observed on infected tomato plants, characterized by small, circular to irregular brown lesions with yellow halos that expanded and coalesced under high humidity (**Figure 1A**). Diseased leaf tissues were surface sterilized and cultured on PDA, resulting in consistent fungal growth after 7 days of incubation at 25 ± 2°C. The emerging colonies were initially white, later turning dark brown to olive green with a cottony texture. Microscopic examination of the isolated fungus revealed septate hyphae, branched conidiophores, and muriform conidia with both transverse and longitudinal septa, typical of the genus *Alternaria* (**Figure 1B**). Based on colony morphology and microscopic features, the pathogen was preliminarily identified as *Alternaria* sp.

### 3.2 Phylogenetic identification of the *Alternaria* isolate Alt2 using multilocus sequence analysis

To determine the taxonomic identity of the isolated fungal strain Alt2, a multilocus phylogenetic analysis was performed using concatenated sequences of three genetic markers: the ITS region (acc#PV239501), *tef1* (acc#PX240704), and *RPB2* (acc#PX240706). A maximum likelihood phylogenetic tree was constructed incorporating reference sequences from various *Alternaria* species, and bootstrap support values were calculated based on 500 replicates to assess the reliability of each clade (**Figure 1C**). The results revealed that isolate Alt2 grouped robustly within the *A. alternata* clade, forming a distinct cluster with the reference strain *A. alternata* SRC11rK2f (GCA_001642055), with a bootstrap value of 100%. This strong statistical support confirms the close evolutionary relationship of Alt2 to other *A. alternata* isolates. The *A. alternata* cluster was further distinguished from neighboring species such as *A. viburni*, *A. conjuncta*, and *A. metachromatica*, which formed sister groups with slightly lower support values (96–88%). More distantly related species, including *A. arborescens*, *A. tenuissima*, *A. infectoria*, and *A. solani*, formed separate lineages within the genus, indicating clear phylogenetic divergence. These findings confirm that the Alt2 isolate belongs to the *A. alternata* species complex. The use of multilocus sequence data, particularly *tef1* and *RPB2* alongside ITS, enhanced species-level resolution and provided a robust framework for accurate identification. The phylogenetic tree was rooted using *Fusarium oxysporum* isolate Fo47^T^ as the outgroup, ensuring the proper orientation of evolutionary relationships within the *Alternaria* genus.

### 3.3 Isolation and morphological identification of *Trichoderma* isolates

Four distinct *Trichoderma* isolates, designated Ham34, Ham35, Ham36, and Ham37, were successfully isolated from rhizosphere soil and cultured on PDA. All isolates exhibited the characteristic rapid growth of the genus. However, macroscopic and microscopic examination revealed distinct morphological variations among them, as detailed in **Supplementary Table S1**. Isolate Ham34 presented a classic *Trichoderma* morphology, with a compact, floccose mycelial texture and uniform, circular colony formation. It displayed robust sporulation, evident as distinct white and green concentric rings. In contrast, Ham37 exhibited weaker growth characteristics, characterized by a loose mycelial texture, light sporulation with yellowish-green pigmentation, and an irregular colony margin. Isolates Ham35 and Ham36 exhibited intermediate characteristics, with Ham35 showing clear concentric zones and Ham36 displaying a fluffier texture with paler, less dense sporulation. Microscopic analysis confirmed the identity of all isolates, revealing the defining structures of the genus, including branched conidiophores and flask-shaped phialides. The specific combination of macroscopic features for each isolate is documented in **Supplementary Table S1**, confirming their placement within the *Trichoderma* genus while highlighting their phenotypic diversity.

### 3.4 Antagonistic effect of Trichoderma isolates against A. alternata using the dual culture technique

**Figure 2:**
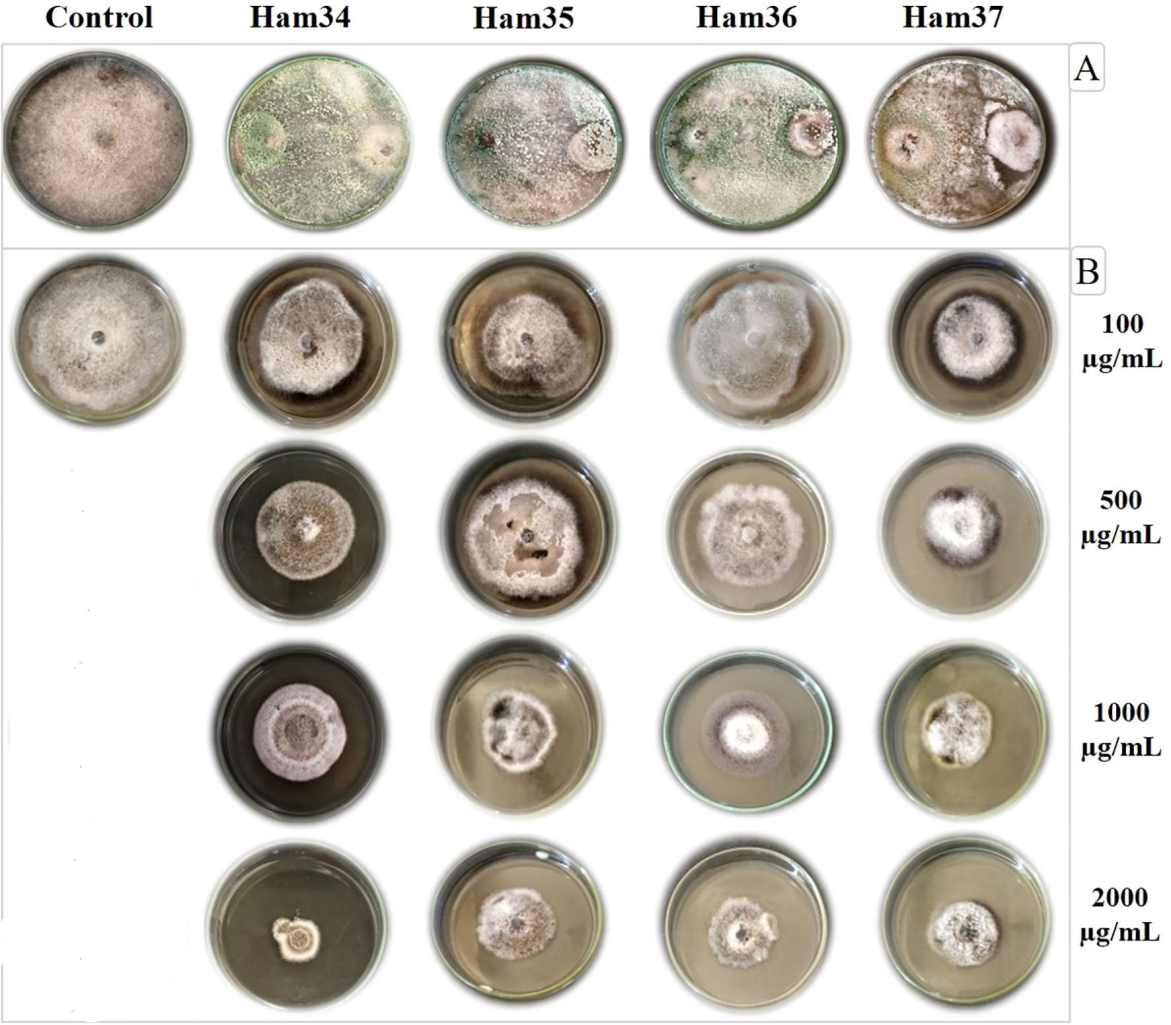
Dual culture assay of *Trichoderma* isolates (Ham34–Ham37) against *Alternaria alternata* compared to the untreated control **(A)** and ethyl acetate extracts of four *Trichoderma* isolates at different concentrations (100, 500, 1000, and 2000 µg/mL) against the growth of *A*. *alternata***(B)**. On plates with dual cultures, *Trichoderma* sp. is on the left, while *Alternaria* sp. is on the right side of the plate.

The dual-culture assay demonstrated that all *Trichoderma* isolates significantly inhibited the growth of *A. alternata* isolate Alt2 compared with the untreated control (**Figure 2A**). One-way ANOVA revealed a significant difference among the isolates (*p* < 0.0001). The highest growth inhibition was recorded for the isolate Ham34 (76.3^a^ %), followed by Ham36 (74.4^ab^ %), Ham35 (72.6^b^ %), and Ham37 (68.1^c^ %), while the control exhibited no inhibition. The calculated LSD_0.05_ was 3.38, confirming statistically significant differences among the treatments. Pairwise comparisons indicated that Ham34 differed significantly from Ham37 (*p* = 0.0013) and Ham35 (*p* = 0.0425), whereas the difference between Ham34 and Ham36 was not statistically significant (*p* = 0.0874). Statistical grouping (LSD test, *p* ≤ 0.05) thus confirmed that isolate Ham34 possessed the most potent antifungal activity against *A. alternata*.

### 3.5 Concentration-dependent antifungal activity of *Trichoderma* ethyl acetate extract

The ethyl acetate extract of four *Trichoderma* isolates exhibited concentration-dependent inhibition of Alt2 growth (**Table 1**). At the control treatment (0 µg/mL of *Trichoderma* extract), no growth inhibition of the Alt2 isolate was observed. At a concentration of 100 µg/mL, the *Trichoderma* ethyl acetate extracts showed apparent antifungal activity. The Ham34 extract showed the highest inhibition (up to 19.6%), followed by Ham35 (up to 16.3%), Ham36 (14.8%), and Ham37 (13.7%). Although Ridomil Gold^®^ presented a more substantial effect at this level (65.2%), these early inhibitory responses confirmed the potential of *Trichoderma* metabolites even at low concentrations (*p* < 0.001, LSD_0.05_ = 3.58). At 500 µg/mL, the antifungal performance of the *Trichoderma* extracts improved markedly. Ham34 extract exerted 39.6% inhibition, while the other isolates showed inhibition ranging from 33.3% to 38.9%. Ridomil Gold^®^ achieved 72.2% inhibition, maintaining superior efficacy; however, significant differences were evident among the *Trichoderma* isolates (*p* < 0.01, LSD_0.05_ = 2.75). At 1000 µg/mL, the inhibition achieved by the *Trichoderma* extracts further strengthened, with the Ham34 extract recording 51.8% and the remaining extracts ranging from 41.1% to 45.2%. Ridomil Gold^®^ maintained the highest inhibition level (82.2%) at this concentration. ANOVA confirmed that treatment effects were highly significant (*p* < 0.001, LSD_0.05_ = 4.42). At the highest tested concentration of 2000 µg/mL, the ethyl acetate extracts showed the highest inhibitory effect. Ham34 extract achieved 57.8% inhibition, while Ham37 extract recorded 52.6%, indicating strong antifungal potential across all isolates. Although Ridomil Gold^®^ fungicide exhibited the highest inhibition (87.1%), the *Trichoderma* extracts demonstrated substantial suppression of fungal growth at this concentration. Statistical analysis showed significant differences among treatments (*p* < 0.001, LSD_0.05_ = 3.87), confirming that all *Trichoderma* isolates exerted substantial and concentration-dependent inhibition of *A. alternata*.

**Table 1:** Comparison of *in vitro* antifungal activity of ethyl acetate extracts from *Trichoderma* isolates and plant extracts versus Ridomil Gold^®^ fungicide against *A. alternata* at different concentrations.

Fungal growth inhibition (% ± SD, CV%)					
Trichoderma ethyl acetate extract	Concentrations (µg/mL)				
	0	100	500	1000	2000
Ham34	0.0^a^ ± 0.00 (0.0)	19.6^b^ ± 2.79 (14.2)	39.6^b^ ± 2.31 (5.8)	51.8^b^ ± 1.69 (3.3)	57.8^b^ ± 2.22 (3.8)
Ham35	0.0^a^ ± 0.00 (0.0)	16.3^bc^ ± 0.64 (3.9)	37.8^b ±^1.11 (2.9)	45.2^b^ ± 4.49 (9.9)	54.8^bc^ ± 2.31 (4.2)
Ham36	0.0^a^ ± 0.00 (0.0)	14.8^c^ ± 1.28 (8.6)	38.9^b^ ± 0.23 (0.6)	44.1^c^± 0.64 (1.5)	54.8^bc ±^1.28 (2.3)
Ham37	0.0^a^ ± 0.00 (0.0)	13.7^c^ ± 1.71 (12.5)	33.3^c^ ± 0.12 (0.4)	41.1^c^ ± 2.22 (5.4)	52.6^c ±^2.79 (5.3)
Ridomil Gold^®^ fungicide	0.0^a^ ± 0.00 (0.0)	65.2^a^ ± 2.56 (3.9)	72.2^a^ ± 2.22 (3.1)	82.2^a ±^1.11 (1.4)	87.1^a ±^1.69 (1.9)
LSD_0.05_	0.00	3.58	2.75	4.42	3.87
Plant extract					
*Rumex dentatus* (RD)	0.0^a^ ± 0.00 (0.0)	33.3^b^ ± 0.00 (0.0)	41.5^b^ ± 2.79 (6.7)	53.7^b^ ± 3.21 (6.0)	68.5^b^ ± 3.20 (4.7)
*Cichorium intybus* L. (CI)	0.0^a^ ± 0.00 (0.0)	29.3^b^ ± 3.56 (12.1)	31.9^c^ ± 2.56 (8.0)	46.7^c^ ± 2.94 (6.3)	62.9^bc^ ± 6.41 (10.2)
*Conium maculatum* (CM)	0.0^a^ ± 0.00 (0.0)	28.9^b^ ± 4.00 (13.8)	27.1^d^ ± 1.28 (4.7)	40.7^d^ ± 3.20 (7.9)	59.3^c^ ± 6.41 (10.8)
*Capsicum annuum* (CA)	0.0^a^ ± 0.00 (0.0)	27.1^b ±^7.80 (28.8)	31.9^c^ ± 1.69 (5.3)	36.7^d^ ± 1.11 (3.0)	56.3^c^ ± 4.49 (8.0)
Ridomil Gold^®^ fungicide	0.0^a^± 0.00 (0.0)	65.2^a^ ± 2.56 (3.9)	72.2^a^ ± 2.22 (3.1)	82.2^a^ ± 1.11 (1.4)	87.1^a^ ± 1.69 (1.9)
LSD_0.05_	0.00	7.98	3.97	4.57	8.74

Data are presented as the mean percentage of fungal growth inhibition ± standard deviation (SD), with CV (%) in parentheses. Means within each column followed by different letters differ significantly (LSD, *p* ≤ 0.05) for each concentration.

### 3.6 Molecular identification of *Trichoderma* isolate Ham34

**Figure 3:**
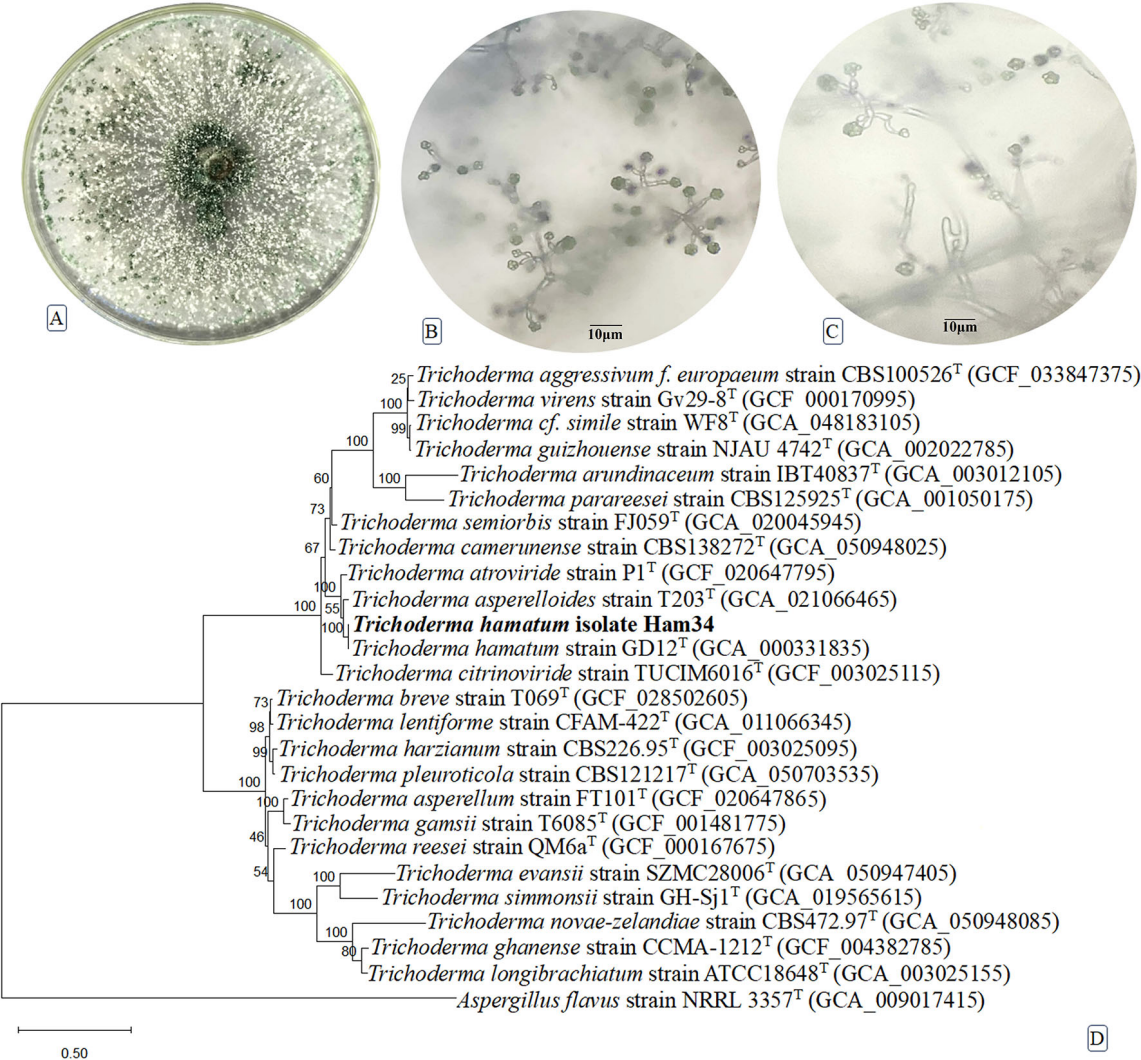
Morphological and growth characteristics of *Trichoderma hamatum* on PDA plate **(A)** showing colony morphology (scale bar = 1 cm); microscopic features of branched conidiophores and flask-shaped phialides bearing single-celled green conidia **(B, C)** (scale bar = 10 µm); maximum-likelihood phylogenetic tree illustrates the relationship of the *Trichoderma hamatum* isolate Ham34 (in bold) with other type strains within the genus *Trichoderma*, derived from a concatenation of three genes: ITS, *RPB2*, and *tef1***(D)**. *Aspergillus flavus*, a member of the genus *Aspergillus*, was used as an outgroup taxon for phylogenetic inference. The partial deletion method was used to remove all positions with less than 95% site coverage, resulting in a final dataset of 2,521 positions. Bootstrap values were derived from 500 replicates using the optimal model (Kimura 2-parameter +*G* +*I*) in MEGA12 software, which utilized up to four parallel computing threads. The tree scale bar represents 0.5 changes per nucleotide position. Type strains (T) are indicated in superscript.

The *Trichoderma* isolate exhibiting the most potent antifungal activity in the *in vitro* assays was designated Ham34. The morphological and growth characteristics of *Trichoderma* on PDA plates (**Figure 3A**) showed a typical colony morphology, accompanied by microscopic features of branched conidiophores and flask-shaped phialides bearing single-celled, green conidia (**Figures 3B, C**). To confirm its taxonomic identity, molecular characterization was performed using concatenated sequences of the ITS, *tef1*, and *RBP2* markers. Based on the combined dataset, phylogenetic analysis placed Ham34 within the genus *Trichoderma*. A maximum-likelihood phylogenetic tree was constructed using concatenated sequences from closely related *Trichoderma* species retrieved from GenBank (**Figure 3**). Phylogenetic analysis clustered Ham34 (ITS: PX024511; *tef1*: PX240702; *RPB2*: PX240708) within a strongly supported clade containing *T. hamatum* isolates. Ham34 grouped closely with reference strains, including *T. hamatum* GD12 (GCA_000331835), with a substantial bootstrap support value of 100%, confirming the precise placement of Ham34 within the *T. hamatum* lineage. Adjacent to this cluster, a separate, well-supported clade of *T. asperelloides* and *T. atroviride* isolates was observed, indicating their close genetic relationship to *T. hamatum*. More distantly, other species such as *T. harzianum*, *T. ghanense*, and *T. longibrachiatum* formed independent clusters, each supported by high bootstrap values, highlighting their genetic divergence from the *T. hamatum* complex. The phylogenetic tree was rooted using *Aspergillus flavus* strain NRRL 3357^T^ as an outgroup, providing clear resolution of the evolutionary relationships among the analyzed taxa. The overall topology, supported by high bootstrap values across the central nodes, provided robust evidence that the isolate Ham34 is a true representative of *T. hamatum* (**Figure 3**).

### 3.7 GC-MS-based chemical profiling of *T. hamatum* Ham34 extract

GC-MS analysis of the ethyl acetate filtrate of *T. hamatum* Ham34 revealed eight major compounds (**Table 2**). The most abundant compounds were (-)-spathulenol (28.2%), glycerol 1,2-diacetate (21.4%), and 4H-1-benzopyran-4-one, 2-(3,4-dimethoxyphenyl)-3,5-dihydroxy-7-methoxy- (10.8%). Other constituents included 3-Amino-4-[(1-benzyl-2-methoxy-2-oxoethyl)amino]-4-oxobutanoic acid (8.4%), 3-buten-2-one, 4-(2,6,6-trimethyl-1-cyclohexen-1-yl)- (7.4%), hexadecanoic acid, methyl ester (3.6%), 11-octadecenal (3.4%), and ethylene brassylate (3.2%). The identified compounds varied in chemical nature, including alcohols, esters, aldehydes, and terpenoids. The GC–MS chromatogram (**Supplementary Figure S1**) is provided in the **Supplementary Materials**.

**Table 2:** Volatile and semi-volatile compounds identified in the ethyl acetate filtrate of *T. hamatum* Ham34 using GC-MS.

RT	Compound	MF	Area (%)	Molecular formula
27.68	Ethylene brassylate	758	3.2	C_15_H_26_O_4_
32.10	11-Octadecenal	716	3.4	C_18_H_34_O
26.21	Hexadecanoic acid, methyl ester	717	3.6	C_17_H_34_O_2_
16.41	3-buten-2-one, 4-(2,6,6-trimethyl-1-cyclohexen-1-yl)-	825	7.4	C_13_H_20_O
35.03	3-Amino-4-[(1-benzyl-2-methoxy-2-oxoethyl)amino]-4-oxobutanoic acid	646	8.4	C_14_H_18_N_2_O_5_
43.55	4H-1-benzopyran-4-one, 2-(3,4-dimethoxyphenyl)-3,5-dihydroxy-7-methoxy-	728	10.8	C_18_H_16_O_7_
13.09	Glycerol 1,2-diacetate	937	21.4	C_7_H_12_O_5_
18.69	(-)-Spathulenol	907	28.2	C_15_H_24_O

RT, Retention Time; MF, Match Factor; Area (%): Relative abundance; Molecular Formula.

### 3.8 Evaluation of plant extracts for inhibition of *A. alternata* growth under *in vitro* conditions

**Figure 4:**
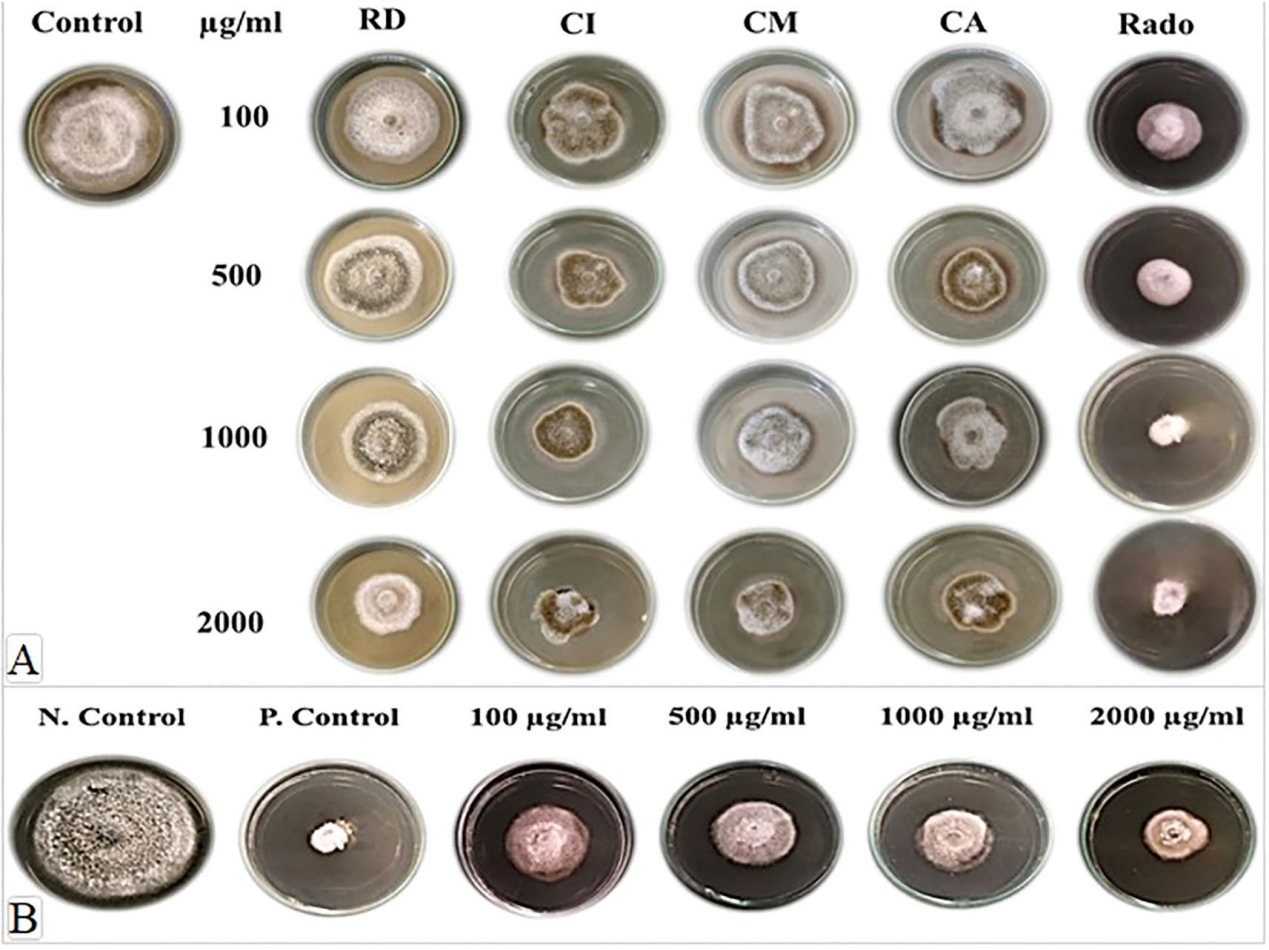
**(A)** Antifungal activity of four plant extracts — *Rumex dentatus* (RD), *Cichorium intybus* (CI), *Conium maculatum* (CM), and *Capsicum annuum* (CA) — against *Alternaria alternata in vitro* at concentrations ranging from 100 to 2000 µg/mL, alongside Ridomil Gold^®^ fungicide (Rado) and the untreated control (0 µg/mL). **(B)** Growth inhibition of *A*. *alternata* by the combined ethyl acetate extract of *Trichoderma hamatum* and *R. dentatus* at different concentrations. N. Control: negative control (*A. alternata* only); P. Control: positive control (Ridomil Gold^®^ fungicide at 2000 µg/mL). Scale bar = 1 cm.

The antifungal activity of four plant extracts —RD, CI, CM, and CA —was evaluated *in vitro* against Alt2 at concentrations ranging from 100 to 2000 µg/mL, compared with a Ridomil Gold^®^-treated control (**Table 1** and **Figure 4**). Among the extracts, RD exhibited the highest inhibitory effect across all concentrations. At 100 µg/mL, the RD extract showed the highest inhibition (33.3%), followed by CI (29.3%), CM (28.9%), and CA (27.1%). In contrast, Ridomil Gold^®^ exhibited significantly higher inhibition (65.2%). Analysis of variance indicated a significant treatment effect (*p* < 0.001, LSD_0.05_ = 7.98). At 500 µg/mL, the antifungal activity increased in all extracts, with RD achieving 41.5% inhibition, while CI, CM, and CA ranged from 27.1% to 31.9%. Ridomil Gold^®^ maintained the highest inhibition (72.2%). The observed variations were statistically significant (*p* < 0.001, LSD_0.05_ = 3.97). At 1000 µg/mL, inhibition continued to rise, with RD recording 53.7%, followed by CI (46.7%), CM (40.7%), and CA (36.7%). Ridomil Gold^®^ displayed 82.2% inhibition. The treatment effect remained highly significant (*p* < 0.0001, LSD_0.05_ = 4.57). At the highest concentration (2000 µg/mL), all extracts reached peak activity. RD extract showed the most potent inhibition (68.5%), followed by CI (62.9%), CM (59.3%), and CA (56.3%). Ridomil Gold^®^ exhibited the most significant inhibition (87.1%). The statistical analysis confirmed significant differences among treatments (*p* < 0.0001, LSD_0.05_ = 8.74).

### 3.9 Quantitative HPLC analysis of bioactive compounds in antifungal *R. dentatus* leaf extract

The plant extract exhibiting the most potent antifungal activity against Alt2, recognized as RD leaf extract, was subjected to HPLC analysis. A total of 16 compounds belonging to the polyphenolic and flavonoid categories were identified (**Table 3**). Among them, rutin was the most abundant, with a concentration of 33.12 µg/mL, followed by gallic acid (22.7 µg/mL) and chlorogenic acid (17.98 µg/mL). Moderate concentrations were observed for ellagic acid (7.54 µg/mL), catechin (4.72 µg/mL), caffeic acid (4.67 µg/mL), and ferulic acid (4.53 µg/mL), as shown in **Table 3**. Compounds such as methyl gallate, syringic acid, vanillin, daidzein, naringenin, and coumaric acid were present in lower amounts. Several compounds, including pyrocatechol, apigenin, and hesperidin, were not detected in the extract. The total quantified polyphenolic and flavonoid content amounted to 108.63 µg/mL (**Table 3**). The HPLC chromatogram of the identified compounds is provided in the **Supplementary Materials** (**Supplementary Figure S2**).

**Table 3:** HPLC profile of bioactive phenolics and flavonoids in *Rumex dentatus* leaves.

Compounds	Retention time	Concentration (µg/mL)
Pyro catechol	6.799	ND
Apigenin	14.50	ND
Hesperidin	15.59	ND
Cinnamic acid	14.03	0.17
Kaempferol	14.97	0.25
Vanillin	9.757	0.83
Naringenin	10.59	0.97
Querectin	12.69	1.20
Coumaric acid	9.156	1.46
Daidzein	0.117	1.77
Methyl gallate	5.646	2.78
Syringic acid	6.583	3.94
Ferulic acid	10.071	4.53
Caffeic acid	6.051	4.67
Catechin	4.674	4.72
Ellagic acid	8.441	7.54
Chlorogenic acid	4.132	17.98
Gallic acid	3.383	22.70
Rutin	7.97	33.12
Total		108.63

ND, Not detected.

### 3.10 Effect of the combination of *R. dentatus* extract and ethyl acetate extract of *T. hamatum* isolate Ham34 on *A. alternata* growth

The inhibition percentage of *A. alternata* growth in culture increased progressively with higher concentrations of the combined RD extract and ethyl acetate extract of Ham34 (**Figure 4B**). The lowest inhibition was observed at 100 µg/mL (53.3%). In comparison, the highest inhibition among the tested treatments occurred at 2000 µg/mL (66.7%). At 1000 µg/mL, the inhibition percentage was recorded as 60.7%. The positive control (Ridomil Gold^®^ fungicide at 2000 µg/mL) showed the most significant inhibition (87.1%). Statistical analysis indicated significant differences between treatments, with an LSD_0.05_ value of 2.63.

### 3.11 Disease incidence and severity of *A. alternata* caused leaf spot on tomato plants under greenhouse conditions

**Figure 5:**
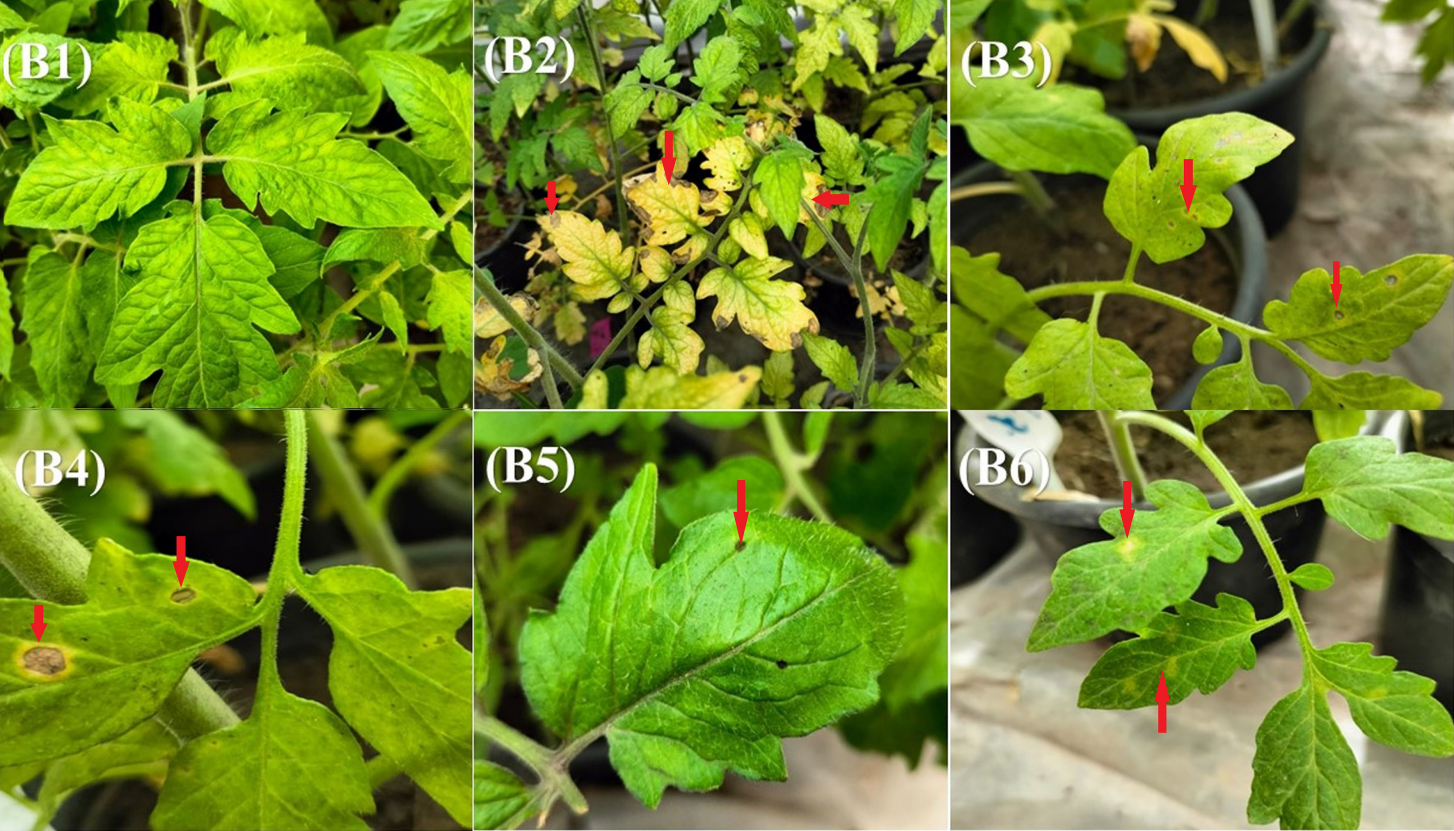
Impact of various treatments on disease incidence and severity in *Alternaria*-infected plants under greenhouse conditions. B1 – healthy untreated control plants; B2 – plants inoculated with *Alternaria* pathogen only; B3 – plants sprayed with plant extract (RD) 48 hours after *Alternaria* inoculation; B4 – plants treated with ethyl acetate extract of *Trichoderma* isolate Ham34–48 hours after pathogen inoculation; B5 – plants treated with a mixture of RD extract and Ham34 ethyl acetate extract 48 hours after inoculation and B6 – plants treated with Ridomil Gold^®^ fungicide 48 hours after *Alternaria* inoculation. The red arrows indicate the leaf spot symptoms caused by *Alternaria* in the different treatments.

To evaluate the effectiveness of various treatments against Alt2 infection, disease incidence and severity were recorded for six combinations. The treatments included untreated controls, pathogen-only inoculation, treatment with RD extract, treatment with Ham34 extract, their combination, and a chemical fungicide (Ridomil Gold^®^). Data are presented in **Table 4**, and statistical significance was assessed using LSD at *p* ≤ 0.05. The uninoculated plants (B1) showed no signs of disease, confirming that the experimental setup was free from contamination (**Figure 5**). In contrast, the untreated, pathogen-inoculated plants (B2) exhibited 100% disease incidence and high disease severity (80.6%), validating the pathogenicity of the Alt2 isolate used (see **Supplementary Materials**, **Supplementary Figure S3**). Application of the plant extract RD (B3) 48 hours post-inoculation resulted in a substantial reduction in disease incidence (22.2%) and severity (16.7%) compared to the untreated, pathogen-only inoculated plants (B2) (*p* = 0.0001 and *p* < 0.0001, respectively). This indicates moderate protective activity of the RD extract. The ethyl acetate extract of Ham34 (B4) also reduced disease parameters, with disease incidence and severity reaching 44.4% (*p* = 0.0010) and 33.3% (*p* = 0.0002), respectively. Although less effective than RD alone, it still provided a significant reduction compared to the untreated infected group (**Table 4**). Interestingly, the combined application of RD extract and Ham34 extract (B5) showed the most potent curative effect, reducing disease incidence to 11.1% (*p* < 0.0001) and severity to 16.7% (*p* < 0.0001). These values were below the LSD threshold, indicating a statistically significant reduction of the symptoms when compared with untreated, inoculated with Alt2 plants (B2), and even slightly better performance than RD alone (B3) or Ridomil Gold^®^ (B6). The chemical fungicide Ridomil Gold^®^ (B6) was effective in reducing disease incidence (22.2%, *p* = 0.0001) and severity (47.2%, *p* = 0.0075). However, its performance was slightly inferior to the combined natural treatment (B5), particularly in terms of disease severity (**Table 4**).

**Table 4:** Effect of various treatments on disease incidence and severity in *Alternaria*-infected plants.

Treatments	Disease incidence (% ± SD, CV%)	Disease severity (% ± SD, CV%)
B1	0.00^c^ ± 0.00 (0.0)	0.00^e^ ± 0.00 (0.0)
B2	100 ^a^ ± 0.00 (0.0)	80.5^a^ ± 4.80 (6.0)
B3	22.2^bc^ ± 19.2 (86.5)	16.6^d^ ± 8.33 (50.1)
B4	44.4^b^ ± 19.2 (43.2)	33.3^c^ ± 8.33 (25.0)
B5	11.1^c^ ± 19.2 (173.0)	16.7^d^ ± 8.33 (49.9)
B6	22.2^bc^ ± 19.2 (86.5)	47.2^b^ ± 4.81 (10.2)
LSD	27.9	11.6

*Data are presented as mean ± standard deviation (SD), with CV (%) in parentheses. Means within each column followed by different letters differ significantly (LSD, *p* ≤ 0.05) for each concentration. B1 – healthy untreated control plants; B2 – plants inoculated with *Alternaria* pathogen only; B3 – plants sprayed with plant extract (RD) 48 hours after *Alternaria* inoculation; B4 – plants treated with ethyl acetate extract of *Trichoderma* isolate Ham34–48 hours after pathogen inoculation; B5 – plants treated with a mixture of RD extract and Ham34 ethyl acetate extract 48 hours after inoculation and B6 – plants treated with Ridomil Gold^®^ fungicide 48 hours after *Alternaria* inoculation.

### 3.12 Plant growth and physiological parameters in response to treatments

The impact of various treatments on plant growth and physiological performance was evaluated based on shoot and root fresh weight, dry weight, length, and chlorophyll content (SPAD value). The results are summarized in **Table 5**, and statistical analysis by one-way ANOVA revealed that all parameters were significantly influenced by treatment (*p* < 0.001 for all traits). In the healthy control group (B1), the shoot and root fresh weights were recorded as 7.92 g and 6.41 g, respectively, while the corresponding dry weights reached 2.66 g for shoots and 1.08 g for roots. Shoot and root lengths were 60.7 cm and 24 cm, respectively, and the chlorophyll content (SPAD value) was 21.7%. Upon inoculation with Alt2 without any treatment (B2), a marked reduction was observed in all growth parameters (*p* < 0.001). Shoot and root fresh weights declined to 7.86 g and 4.18 g, while dry weights dropped to 2.56 g and 0.61 g, respectively. Similarly, shoot and root lengths decreased to 61.7 cm and 20.3 cm, and chlorophyll content dropped sharply to 10.1%, reflecting the pathogen’s adverse effect on plant growth and photosynthetic activity (**Table 5**). Treatment with the RD extract alone (B3) significantly improved plant growth compared with the infected control (*p* < 0.001). Shoot and root fresh weights increased to 22.8 g and 12.8 g, while dry weights reached 4.89 g and 3.01 g, respectively. Shoot and root lengths extended to 83 cm and 36 cm, and the chlorophyll SPAD value rose to 29.2%. In the Ham34 extract treatment (B4), the shoot and root fresh weights were 18.4 g and 9.57 g, respectively, while the dry weights were 3.91 g and 2.33 g, respectively. Shoot and root lengths reached 65.3 cm and 35.7 cm, and chlorophyll content was 27.1%. The combined RD + Ham34 treatment (B5) resulted in further enhancement across all growth parameters. Shoot and root fresh weights were 18.9 g and 11.9 g, while dry weights reached 4.79 g and 2.64 g. Shoot and root lengths were 70 cm and 35.7 cm, and chlorophyll content increased to 29.1%. Treatment with the chemical fungicide Ridomil Gold^®^ (B6) produced moderate responses compared with the untreated infected control, with shoot and root fresh weights of 15.4 g and 8.78 g, dry weights of 3.41 g and 1.10 g, shoot and root lengths of 65 cm and 26 cm, and chlorophyll content of 24.7% (**Table 5**).

**Table 5:** Effect of different treatments on plant biomass, length, chlorophyll content (SPAD), activities of catalase (CAT), peroxidase (POD), superoxide dismutase (SOD), polyphenol oxidase (PPO), malondialdehyde (MDA), and hydrogen peroxide (H_2_O_2_) in tomato leaves, as well as total protein (TP) and total phenolic compounds (TPC), under greenhouse conditions upon *Alternaria* infection.

Parameter value		B1	B2	B3	B4	B5	B6	LSD
Fresh weight (g)	>Shoots	7.92^c^ ± 1.75 (22.1)	7.86^c^ ± 1.99 (25.3)	22.8^a^ ± 5.84 (25.6)	18.4^ab^ ± 1.44 (7.8)	18.9^ab^ ± 2.03 (10.7)	15.4^b^ ± 1.35 (8.8)	5.09
	Roots	6.41^de^ ± 2.19 (34.2)	4.18^e^ ± 0.42 (10.0)	12.8^a^ ± 1.06 (8.3)	9.57^bc^ ± 1.74 (18.2)	11.9^ab^ ± 1.16 (9.7)	8.78^cd^ ± 2.78 (31.7)	3.10
Dry weight (g)	Shoots	2.66^c^ ± 1.07 (40.2)	2.56^c^ ± 0.72 (28.1)	4.89^a^ ± 0.34 (7.0)	3.91^ab^ ± 0.11 (2.8)	4.79^a^ ± 0.55 (11.5)	3.41^bc^ ± 0.72 (21.1)	1.18
	Roots	1.08^c^ ± 0.20 (18.5)	0.61^d^ ± 0.11 (18.0)	3.01^a^ ± 0.09 (3.0)	2.33^b^ ± 0.32 (13.7)	2.64^b^ ± 0.12 (4.5)	1.10^e^ ± 0.10 (9.1)	0.32
Length (cm)	Shoots	60.7^d^ ± 1.15 (1.9)	61.7^d^ ± 1.15 (1.9)	83^a^ ± 2.64 (3.2)	65.3^c^ ± 2.51 (3.8)	70 ^b^ ± 1.01 (1.4)	65^d^ ± 1.00 (1.5)	3.08
	Roots	24^bc^ ± 3.60 (15.0)	20.3^c^ ± 5.03 (24.8)	36^a^ ± 1.73 (4.8)	35.7^a^ ± 2.30 (6.4)	35.7^a^ ± 1.15 (3.2)	26^b^ ± 1.73 (6.7)	5.18
Chlorophyll SPAD %		21.7^c^ ± 1.52 (7.0)	10.1^d^ ± 1.11 (11.0)	29.2^a^ ± 1.82 (6.2)	27.1^ab^ ± 2.26 (8.3)	29.1^a^ ± 1.70 (5.8)	24.7^b^ ± 0.46 (1.9)	2.84
CAT (μM/g FW)		6.58^a^ ± 0.28 (4.3)	1.31^c^ ± 0.06 (4.6)	0.73^e^ ± 0.03 (4.1)	1.05^d^ ± 0.02 (1.9)	2.02^b^ ± 0.02 (1.0)	0.55^e^ ± 0.04 (7.3)	0.22
POD (μM/g FW)		4.28^d^ ± 0.02 (0.5)	5.53^c^ ± 0.04 (0.7)	2.78^e^ ± 0.01 (0.4)	8.04^a^ ± 0.04 (0.5)	5.78^b^ ± 0.01 (0.2)	2.42^f^ ± 0.03 (1.2)	0.06
SOD (μM/g FW)		195.2^ab^ ± 4.14 (2.1)	200.6^a^ ± 1.08 (0.5)	193.6^ab^ ± 13.4 (6.9)	185.6^b^ ± 10.6 (5.7)	200.3^a^ ± 3.52 (1.8)	192.1^ab^ ± 2.97 (1.5)	13.2
PPO (μM/g FW		0.27^b^ ± 0.00 (0.0)	0.22^f^ ± 0.08 (36.4)	0.24^d^ ± 0.08 (33.3)	0.26^c^ ± 0.08 (30.8)	0.24^e^ ± 0.08 (33.3)	0.27^a^ ± 0.08 (29.6)	8.38
MDA (μM/g FW)		0.57^e^ ± 0.00 (0.0)	1.02^a^ ± 0.02 (2.0)	0.61^d^ ± 0.00 (0.0)	0.71^c^ ± 0.00 (0.0)	0.73^b^ ± 0.01 (1.4)	0.60^d^ ± 0.00 (0.0)	0.02
H_2_O_2_ (μM/g FW)		0.16^e^ ± 0.00 (0.0)	0.24^b^ ± 0.00 (0.0)	0.22^d^ ± 0.061 (27.7)	0.23^c^ ± 0.061 (26.5)	0.22^d^ ± 0.061 (27.7)	0.24^a^ ± 0.00 (0.0)	5.81
Protein (μg/mL)		331.1^c^ ± 0.00 (0.0)	325.8^d^ ± 0.00 (0.0)	325.1^d^ ± 0.30 (0.09)	316.7^e^ ± 0.30 (0.09)	342.8^a^ ± 0.60 (0.18)	332.8^b^ ± 0.80 (0.24)	0.79
TPC (mg GAE/g)		58.9^f^ ± 0.36 (0.61)	145.8^e^ ± 0.36 (0.25)	161^d^ ± 0.62 (0.39)	250.4^b^ ± 1.08 (0.43)	256^a^ ± 0.02 (0.01)	186^c^ ± 0.00 (0.0)	0.98

Data are expressed as mean ± standard deviation (SD) with CV (%) in parentheses. Means within the same row followed by different letters are significantly different according to the least significant difference (LSD) test at *p* ≤ 0.05. B1 – healthy untreated control plants; B2 – plants inoculated with *Alternaria* pathogen only; B3 – plants sprayed with plant extract (RD) 48 hours after *Alternaria* inoculation; B4 – plants treated with ethyl acetate extract of *Trichoderma* isolate Ham34–48 hours after pathogen inoculation; B5 – plants treated with a mixture of RD extract and Ham34 ethyl acetate extract 48 hours after inoculation and B6 – plants treated with Ridomil Gold® fungicide 48 hours after *Alternaria* inoculation.

### 3.13 Antioxidant enzyme activities, oxidative stress markers, total protein assay, and total phenolic content

The CAT activity was highest in the healthy, untreated control (B1, 6.58 μM/g FW) and significantly greater than in all other treatments (*p* < 0.001). Among treated groups, B5 treatment exhibited the second-highest CAT activity (2.02 μM/g FW), followed by B2 (1.31 μM/g FW), B4 (1.05 μM/g FW), and B3 (0.73 μM/g FW), while Ridomil Gold^®^ (B6, 0.55 µM g^−1^ FW) exhibited the lowest activity. POD activity also varied significantly among treatments (*p* < 0.001). The Ham34 extract (B4) produced the highest POD activity (8.04 μM/g FW), followed by the combined extract (B5, 5.78 μM/g FW), the pathogen-only control (B2, 5.53 μM/g FW), B1 (4.28 μM/g FW), B3 (2.78 μM/g FW), and B6 (2.42 μM/g FW). SOD activity did not differ significantly across treatments (*p* = 0.208), remaining within a narrow range (185.6-200.6 μM/g FW). PPO activity showed strong treatment effects (*p* < 0.001), with the highest activity in both the healthy control (B1) and Ridomil Gold^®^ treatment (B6) (0.27 µM/g FW), and the lowest in the pathogen-only control (B2, 0.22 µM/g FW). Whereas B4 (0.26 μM/g FW), B3, and B5 (0.24 μM/g FW) **Table 5**. Malondialdehyde (MDA) levels, an indicator of lipid peroxidation, differed significantly (*p* < 0.001), being lowest in the healthy untreated plants (B1, 0.57 μM/g FW), followed by B6 (0.60 μM/g FW), B3 (0.61 μM/g FW), B4 (0.71 μM/g FW), B5 (0.73 μM/g FW), and B2 (1.02 μM/g FW), which recorded the highest MDA value. All treatments reduced MDA accumulation relative to the infected control. Hydrogen peroxide (H_2_O_2_) accumulation varied significantly (*p* < 0.001). Healthy untreated plants (B1) exhibited the lowest content (0.16 µM/g FW), whereas the pathogen-inoculated control (B2) and Ridomil Gold^®^ treatment (B6) showed the highest levels (0.24 µM/g FW). Intermediate values (0.22–0.23 µM/g FW) were recorded in plants treated with RD extract (B3), Ham34 extract (B4), and their combination (B5) (**Table 5**). Soluble protein content varied significantly across treatments (*p* < 0.001). The highest protein content was recorded in the plants treated with the combined RD + Ham34 extract (342.8 μg/mL), followed by B6 (332.8 μg/mL), B1 (331.1 μg/mL), B2 (325.8 μg/mL), B3 (325.1 μg/mL), and B4 (316.7 μg/mL), which had the lowest protein content. The TPC was also significantly affected (*p* < 0.001). The combined extract (B5, 256 mg GAE/g) and Ham34 extract (B4, 250.3 mg GAE/g) recorded the highest phenolic levels, followed by B6, B3, and B2 (186, 161, and 145.8 mg GAE/g, respectively), while the lowest TPC occurred in the untreated healthy control (B1, 58.9 mg GAE/g) (**Table 5**).

## 4 Discussion

Biological control presents a promising and environmentally sustainable alternative, particularly in light of the harmful ecological impacts associated with the use of fungicides (Shaili et al., 2025; Villavicencio-Vásquez et al., 2025). Biological control agents, including *Trichoderma* species and plant extracts, have demonstrated considerable effectiveness in hindering the proliferation of phytopathogens through various specialized mechanisms, including parasitism, antibiosis, and induced systemic resistance (Aseel et al., 2025; Khalil et al., 2025; Saini et al., 2025). The present investigation effectively isolated and characterized *A. alternata* as the responsible agent for leaf spot disease in tomato plants. Morphological observations, including the presence of septate hyphae, branched conidiophores, and muriform conidia, were consistent with typical *Alternaria* species characteristics (Simmons, 2007). Further molecular identification using MLSA of ITS, *tef1*, and *RPB2* robustly confirmed the grouping of the isolate Alt2 with the reference strain of *A. alternata*, supported by the highest bootstrap value. This integrative approach ensured precise species-level identification, which is fundamental for implementing effective management strategies (Woudenberg et al., 2015; Dettman et al., 2023).

The results of this study reinforce the well-established potential of *Trichoderma* spp. and plant-derived extracts as effective biological control agents against *A. alternata*. Among the tested isolates, *T. hamatum* (Ham34) demonstrated strong antagonistic potential, consistent with previous reports highlighting the multifaceted biocontrol mechanisms of *Trichoderma*, including mycoparasitism, antibiosis, and induction of host defense responses (Arzanlou et al., 2014; Baazeem et al., 2021). The ethyl acetate extract of *Trichoderma* isolates also exhibited concentration-dependent inhibitory effects, with Ham34 extract showing the most potent antifungal effect at the maximum tested concentration. MLSA confirmed that the most potent isolate, Ham34, belongs to *T. hamatum* and clusters closely with other *T. hamatum* isolates in phylogenetic analysis. *T. hamatum* is known for producing a diverse array of secondary metabolites with antifungal properties (Philip et al., 2024). Its antagonistic potential is likely driven by a complex mixture of bioactive compounds, as confirmed by GC–MS analysis of the ethyl-acetate filtrate from the Ham34 strain, which identified several key metabolites belonging to distinct chemical classes, namely (−)-spathulenol, glycerol 1,2-diacetate, and 4H-1-benzopyran-4-one, 2-(3,4-dimethoxyphenyl)-3,5-dihydroxy-7-methoxy-. Among these, (−)-spathulenol, a tricyclic sesquiterpene alcohol with an azulene-type skeleton, was the most abundant compound.

Spathulenol has been frequently reported as a dominant component of essential oils with strong antimicrobial properties. For instance, the essential oil of *Baccharis dracunculifolia*, which contains approximately 27% spathulenol, exhibits marked fungistatic and fungicidal activity (Cazella et al., 2019). Similarly, spathulenol accounted for 21.36% of *Eugenia calycina* leaf oil, where it inhibited *Prevotella nigrescens* and *Porphyromonas gingivalis* at a minimum inhibitory concentration (MIC) of 100 µg/mL (Sousa et al., 2015). In *Salvia cilicica*, spathulenol (23.8%) and caryophyllene (14.9%), which co-occur, contribute significantly to the antimicrobial effects against *Candida* species (Tan et al., 2016). These findings are consistent with the broader antimicrobial profile of sesquiterpenoids, which are recognized for their diverse structural frameworks and potent antibacterial and antifungal properties (Li et al., 2022a). The lipophilic character of spathulenol facilitates its interaction with lipid bilayers, allowing it to penetrate cellular membranes and reach intracellular targets (Chan et al., 2016). This high hydrophobicity enhances its ability to disturb the integrity of fungal membranes, leading to increased permeability and leakage of essential ions, such as K^+^, from microbial cells (Inoue et al., 2004). Such membrane-disruptive and oxidative stress–inducing effects have been proposed as key mechanisms underlying the antimicrobial activity of many sesquiterpenes. Therefore, the strong antifungal potential previously attributed to spathulenol (Dlugoviet et al., 2025), together with its high abundance in the Ham34 extract, strongly supports its role as a major contributor to the observed inhibition of *A. alternata* growth.

Glycerol 1,2-diacetate, commonly known as diacetin, is the diacetyl ester derived from glycerol. Although it is present at 21.4% in GC-MS, the literature does not provide clear evidence for its efficacy as a potent antifungal metabolite. Most reports treat glycerol or glycerol derivatives as humectants, solvents, or carriers rather than dedicated fungicides. For instance, studies have shown that glycerol may modulate microbial growth or metabolism (Sleem et al., 2023), but no specific antifungal mechanism or activity against phytopathogens has been reported. Therefore, glycerol 1, 2-diacetate likely plays a secondary role, either as a solubility/transport tag for other actives or as a metabolic by-product, rather than as a primary fungicide. 4H-1-Benzopyran-4-one, 2-(3,4-dimethoxyphenyl)-3,5-dihydroxy-7-methoxy- is a substituted chromone/flavone-type scaffold (i.e., a 4H-benzopyran-4-one core bearing methoxy/hydroxy substituents and a dimethoxyphenyl moiety). Flavonoid and chromone derivatives are increasingly recognized as antifungal agents, with numerous reported compounds capable of disrupting fungal spore germination, metabolism, or cell wall synthesis (Pistelli and Giorgi, 2012). The derivatives of chromone additionally emphasize the antibacterial and antifungal potential of chromone hybrids (Mohsin et al., 2020). Thus, the presence of ~10.8 % of this chromone-type metabolite suggests a plausible contribution to antifungal activity, possibly via enzyme inhibition, oxidative stress induction, or synergy with the terpenoid component.

Taken together, the chemical profile of the Ham34 extract suggests a multimodal antifungal mixture rather than a single compound effect. The dominant sesquiterpene (spathulenol) may rapidly act on fungal membranes; the chromone-type compound may provide sustained enzyme- or spore-germination inhibition; and the glycerol ester may assist solubilization/transport of other actives or reflect general metabolic overflow. In the context of *A. alternata*, which is sensitive to antagonistic metabolites from *Trichoderma* spp., this extract may inhibit growth, spore germination, or viability via combined modes. Importantly, because each compound has not yet been individually tested against *A. alternata*, the conclusions must remain provisional: we recommend bioassay-guided fractionation, purification, and MIC/spore germination assays of these compounds (both singly and in combination) to define their relative contributions. In summary, the high abundance of spathulenol and the presence of a chromone-flavone type metabolite in the Ham34 extract align well with the literature on antifungal sesquiterpenoids and flavonoid derivatives. By contrast, the glycerol 1,2-diacetate component lacks a specific antifungal precedent but may serve a supportive role. This compositional insight strengthens the hypothesis that the Ham34 filtrate exerts antifungal effect on *A. alternata* via synergistic, multi-component antibiosis rather than a single “magic” molecule. As a limitation of the present study, the individual antifungal activities of the major compounds identified by GC–MS were not experimentally validated. Future studies will therefore focus on isolating these metabolites and evaluating their efficacy, individually and in combination, against *A. alternata* and other phytopathogenic fungi to confirm their specific roles in the observed antifungal activity.

The ethanolic extract of *R. dentatus* also demonstrated vigorous antifungal activity, consistent with earlier studies that highlighted its potency against various pathogens (Batool et al., 2019; Khaliq et al., 2023). HPLC analysis confirmed that the RD extract was rich in phenolic and flavonoid compounds, predominantly rutin, gallic acid, and chlorogenic acid. Along with ellagic acid, catechin, caffeic acid, and ferulic acid, these metabolites likely contribute to the extract’s antifungal potency through complementary antimicrobial, antioxidant, and elicitor mechanisms (Ma et al., 2007; Seo et al., 2013; El-Nagar et al., 2020; Deresa and Diriba, 2023; Mane et al., 2024). Rutin, a prominent flavonoid identified in RD extract, has been reported to exert antifungal activity against various fungal strains, including *Candida gattii* (Abdelaziz et al., 2023) and *Aspergillus fumigatus* (Li et al., 2025), through mechanisms involving disruption of membrane integrity and interference with ergosterol biosynthesis (Han, 2009; Gupta et al., 2024; Li et al., 2025). Moreover, its strong antioxidant capacity may contribute to an imbalance in oxidative stress in fungal cells, further enhancing toxicity (Gullón et al., 2017). Despite the absence of direct MIC data for *A. alternata*, the structural characteristics of rutin and its frequent presence in antifungal plant extracts (Behiry et al., 2022a) support its potential role as a key factor in the observed activity. Gallic acid (3,4,5-trihydroxybenzoic acid) is another key phenolic with extensive evidence of antifungal efficacy. Gallic acid exhibits broad-spectrum antifungal activity against dermatophyte strains (Wu et al., 2009), and its alkyl derivatives significantly inhibited the mycelial growth of *A. solani* and *F. solani*, showing dose-dependent reduction in colony diameter and spore germination (El-Nagar et al., 2020). Studies show that the antifungal effects of gallic acid may be dose-dependent (Seo et al., 2013). Its mode of action has been linked to the generation of ROS, lipid peroxidation, and membrane leakage (Rahimifard et al., 2020). Therefore, the presence of gallic acid in the RD extract strongly supports its role in direct antagonism of *A. alternata*. Chlorogenic acid (5-caffeoylquinic acid) is similarly known for its broad-spectrum antifungal activity. It has been shown to cause membrane permeabilization and intracellular ion leakage in multiple phytopathogenic fungi (Ma et al., 2007; Sung and Lee, 2010; Martínez et al., 2017). More recent research demonstrated chlorogenic acid-induced ROS accumulation and apoptosis in *F. fujikuroi* (Kai et al., 2021), suggesting that its fungicidal effect involves both oxidative and membrane-disruptive mechanisms. Within our results, chlorogenic acid appears to act synergistically with gallic acid and rutin, inhibiting the growth of *A. alternata*.

Secondary phenolics, such as ellagic acid, catechin, caffeic acid, and ferulic acid, were detected at moderate levels and remain relevant due to their known additive or synergistic antifungal properties. Ellagic acid inhibits spore germination and hyphal elongation in *B. cinerea* and *Candida* spp. through oxidative stress induction (Vattem and Shetty, 2005; Navarro-Martínez et al., 2006; Le et al., 2013; Possamai Rossatto et al., 2021). Catechin, a flavan-3-ol, impairs fungal cell walls and inhibits glucan synthase activity (Sitheeque et al., 2009). Caffeic acid and ferulic acid—both hydroxycinnamic derivatives—have been reported to damage fungal membranes and inhibit ergosterol synthesis, reducing viability in *Botrytis, Alternaria*, and *Fusarium* species (Patzke and Schieber, 2018; Possamai Rossatto et al., 2021; Behiry et al., 2022a; Kong et al., 2023). Collectively, these mid-level phenolics are likely to potentiate the effects of major compounds through redox cycling and cell-wall interference. The coexistence of high concentrations of rutin, gallic acid, and chlorogenic acid, along with multiple supporting phenolics, suggests that the RD extract exerts antifungal effects through multi-targeted and synergistic mechanisms, rather than via a single dominant molecule. This mixture likely disrupts fungal membranes, triggers ROS-mediated oxidative damage, and interferes with essential biosynthetic pathways, leading to impaired mycelial growth and spore germination. Such synergy is consistent with earlier reports demonstrating enhanced antifungal efficacy of combined phenolics compared with single-compound treatments (Al Aboody and Mickymaray, 2020; Rodríguez et al., 2023). Future fractionation and MIC assays should clarify the relative contributions of these individual molecules and verify whether the observed inhibition of *A. alternata* results primarily from additive or synergistic interactions.

Prominently, the combination of Ham34 ethyl acetate extract with RD extract under greenhouse conditions resulted in a synergistic reduction in disease incidence and severity, surpassing the effect of the commercial fungicide Ridomil Gold^®^. This integrative effect reflects the complementary modes of action: *Trichoderma* induces host resistance and produces antifungal metabolites. At the same time, *R. dentatus* supplies polyphenolic compounds that directly inhibit the growth of the pathogen. Similar synergistic outcomes have been observed when combining plant extracts with *Trichoderma* species for enhanced disease management (Batool et al., 2019; Khalil et al., 2022; Khaliq et al., 2023). These findings highlight the potential of integrated natural treatments as effective, low-toxicity alternatives to synthetic fungicides. Beyond pathogen suppression, both treatments promoted plant growth and enhanced physiological traits, including shoot and root development, as well as chlorophyll content. This may result from the bioactive compounds in RD acting as biostimulants, as well as from Ham34’s ability to solubilize nutrients, produce phytohormones, and induce plant defense responses (Harman et al., 2004; Batool et al., 2019; Saldaña-Mendoza et al., 2023) The combined treatment of RD extract and Ham34 also resulted in enhanced plant performance, with improvements comparable to or exceeding those of the individual treatments. This synergistic effect on plant growth further supports the potential of this combined approach for holistic plant health management. Enhanced antioxidant enzyme activities (POD and CAT) and reduced oxidative stress markers (MDA and H_2_O_2_) in treated plants indicate that these natural agents not only limit fungal colonization but also strengthen the host’s defense system (Abdelkhalek et al., 2025b; Sheoran et al., 2025).

The present findings highlight the practical potential of integrating Ham34 and RD extracts as eco-friendly, cost-effective alternatives to synthetic fungicides for managing tomato leaf spot. *Trichoderma* species are widely recognized for their biocontrol and plant growth–promoting properties, achieved through mechanisms such as mycoparasitism, antibiosis, and induction of systemic resistance (Yao et al., 2023; Guzmán-Guzmán et al., 2025). Compared with conventional chemical fungicides, this dual-biocontrol approach offers several advantages, including lower production costs due to the easy cultivation and formulation of *Trichoderma* (Woo et al., 2014; Lodi et al., 2023) and the natural abundance of RD, a species known for its rich polyphenolic composition and antimicrobial potential (Nisa et al., 2013; Khaliq et al., 2023). Additionally, both biocontrol agents exhibit high stability and bioactivity under ambient conditions, thereby reducing the need for frequent reapplication (Abdelkhalek et al., 2022a; Li et al., 2022b). The formulation’s biodegradability and non-phytotoxic nature make it particularly suitable for sustainable and organic farming systems (Lamichhane et al., 2016; Teixidó et al., 2021). These characteristics not only minimize environmental contamination and mitigate the development of pathogen resistance but also align with global strategies to replace synthetic agrochemicals with safer, bio-based products (Woo et al., 2014; Feng et al., 2025). Therefore, the combined use of Ham34 and RD represents a promising step toward developing an affordable and environmentally sound biocontrol formulation for large-scale field applications.

Although the present study demonstrated the strong antifungal potential of Ham34 and RD extracts against Alt2, some limitations should be acknowledged. The experiments were conducted primarily under controlled *in vitro* and greenhouse conditions, which may not fully represent the complex interactions that occur under field conditions, where environmental factors and microbial communities can significantly impact performance (El-Hassan et al., 2013; Li et al., 2022b). Therefore, further validation through multi-location field trials is required before large-scale application. Moreover, while GC-MS and HPLC analyses revealed several bioactive compounds in both extracts, the specific contribution of each metabolite to the observed antifungal effect remains unclear. Bioassay-guided fractionation and molecular docking approaches could help identify the most active molecules and their synergistic interactions (Qazi et al., 2022; Li et al., 2023). Future research should also focus on developing optimized formulations with enhanced shelf life and stability, as these aspects are crucial for commercialization and farmer adoption (Villavicencio-Vásquez et al., 2025). Furthermore, assessing potential non-target effects on beneficial soil microorganisms and ecosystem balance remains essential to confirm the environmental safety of these biocontrol agents (Guzmán-Guzmán et al., 2025). Ultimately, future research should focus on large-scale field evaluations, the isolation of key bioactive molecules, the exploration of molecular mechanisms, and the development of formulations. Addressing these aspects will help translate the promising results obtained under controlled conditions into practical and sustainable biocontrol solutions for managing *A. alternata* and other phytopathogenic fungi in agricultural systems.

## 5 Conclusion

The present study demonstrates a practical, eco-friendly approach to managing *A. alternata* leaf spot in tomato plants by combining Rumex dentatus extract with *T. hamatum*. Accurate pathogen identification using multilocus sequencing ensured reliable diagnostics for targeted control. Both *R. dentatus* and *T. hamatum* extracts showed strong antifungal activity *in vitro*, while their combined application provided superior protection under greenhouse conditions, surpassing even the commercial fungicide. Beyond pathogen suppression, these treatments enhanced plant growth, chlorophyll content, and antioxidant defense, reducing oxidative stress. The findings highlight the potential of integrating natural plant-derived and microbial agents as sustainable alternatives to synthetic fungicides. Future studies should investigate the molecular basis of their synergistic action, optimize application strategies, and validate efficacy under field conditions.

## Data Availability

The datasets presented in this study can be found in online repositories. The names of the repository/repositories and accession number(s) can be found in the article/**Supplementary Material**.
