# Supplementary material for: Synergistic curative effects of Trichoderma hamatum and Rumex dentatus against Alternaria alternata, the causal agent of tomato leaf spot disease
Source: Front Plant Sci. 2025 Dec 9;16:1700051. doi: 10.3389/fpls.2025.1700051 (PMC12728584; doi:10.3389/fpls.2025.1700051)
Supplement: Supplementary file 1 [file DataSheet1.docx]

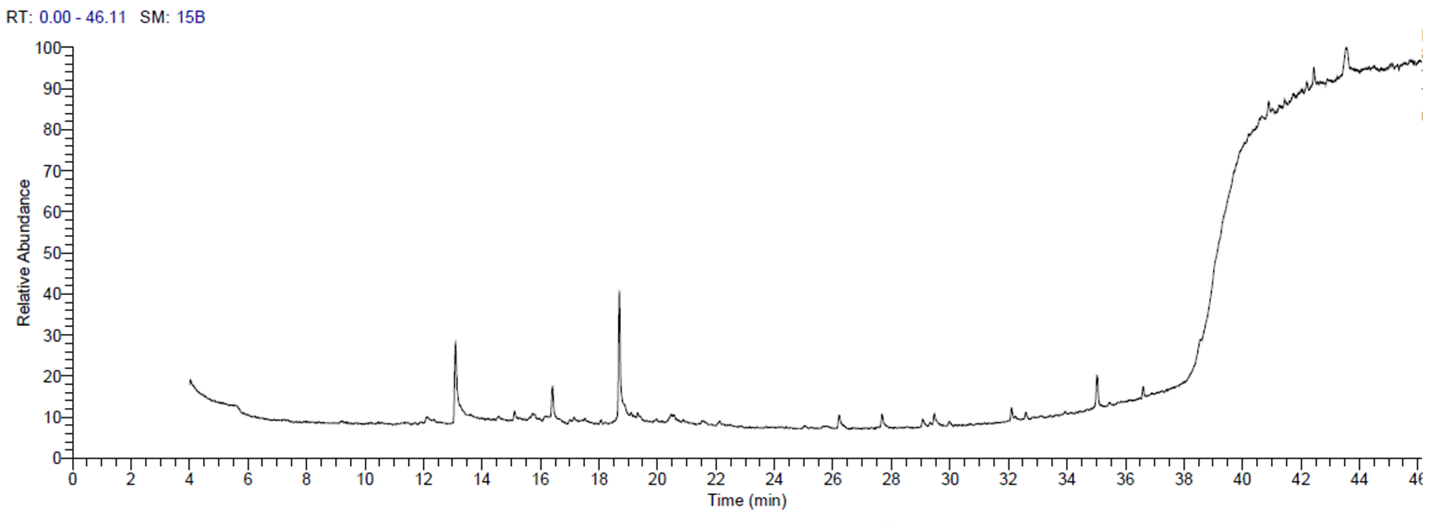


**Figure S1.** Gas chromatography mass spectroscopy profile of *Trichoderma hamatum* ethyl acetate extract.

**
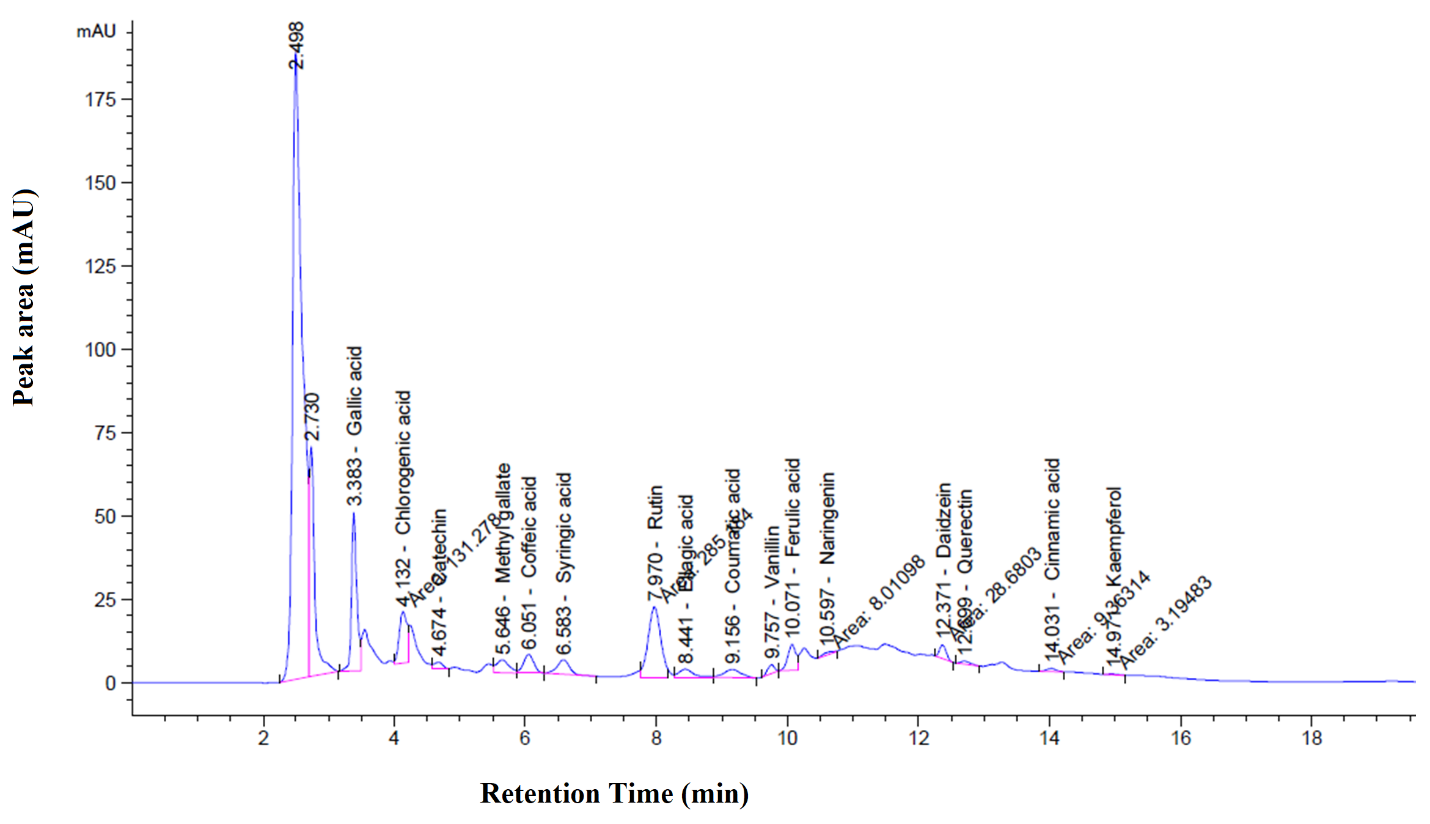
**

**Figure S2**. HPLC chromatogram of *Rumex dentatus* ethanolic extract


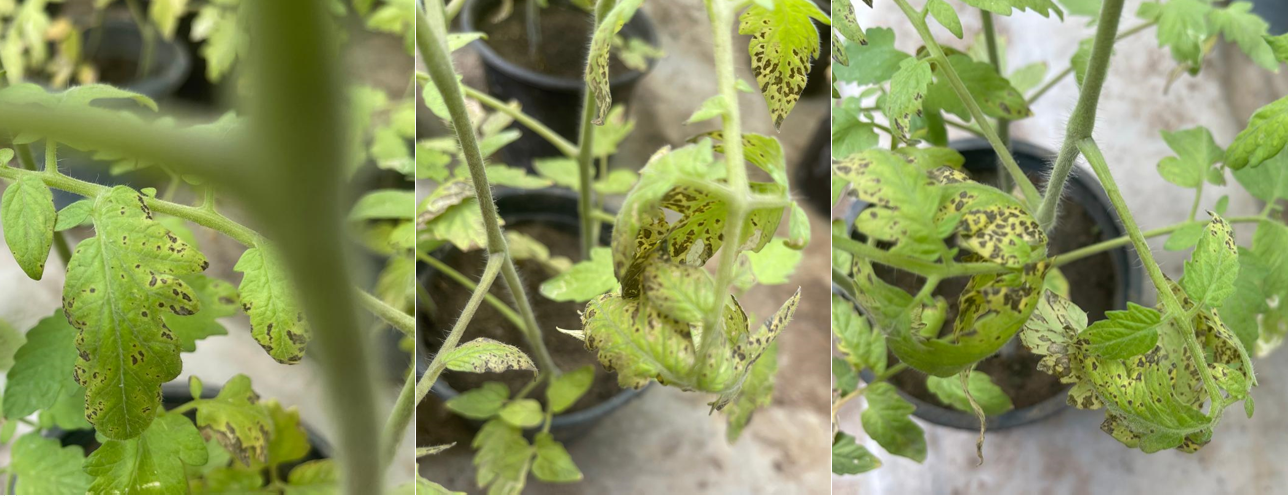


**Figure S3.** Characteristic leaf spot symptoms on tomato cv. ‘Super Strain B’ leaves inoculated with the *Alternaria alternata* pathogen.
