# Supplementary material for: Synergistic curative effects of Trichoderma hamatum and Rumex dentatus against Alternaria alternata, the causal agent of tomato leaf spot disease
Source: Front Plant Sci. 2025 Dec 9;16:1700051. doi: 10.3389/fpls.2025.1700051 (PMC12728584; doi:10.3389/fpls.2025.1700051)
Supplement: Supplementary file 2 [file Table1.docx]

**Table S1.** Morphological characteristics of *Trichoderma* isolates grown on PDA

| **Isolate code** | **Colony color (PDA)** | **Mycelial texture** | **Sporulation intensity** | **Remarks** | **Microscopic features** |
| --- | --- | --- | --- | --- | --- |
| Ham34 | White and Green with patchy, concentric rings of forest-green sporulation. | Compact, floccose | Moderate | Uniform sporulation, circular colony | Branched conidiophores, flask-shaped phialides |
| Ham35 | Green with concentric zones | Slightly cottony | Moderate | Distinct growth rings are visible | Branched conidiophores, flask-shaped phialides |
| Ham36 | Pale green with a faint central zone | Fluffy | Moderate | Less dense sporulation | Branched conidiophores, flask-shaped phialides |
| Ham37 | Yellowish-green with irregular margin | Loose | Light | Weak sporulation and pigmentation | Branched conidiophores, flask-shaped phialides |
